# *Leishmania* blood parasite dynamics during and after treatment of visceral leishmaniasis in Eastern Africa: A pharmacokinetic-pharmacodynamic model

**DOI:** 10.1371/journal.pntd.0012078

**Published:** 2024-04-19

**Authors:** Luka Verrest, Séverine Monnerat, Ahmed M. Musa, Jane Mbui, Eltahir A. G. Khalil, Joseph Olobo, Monique Wasunna, Wan-Yu Chu, Alwin D. R. Huitema, Henk D. F. H. Schallig, Fabiana Alves, Thomas P. C. Dorlo

**Affiliations:** 1 Department of Pharmacy & Pharmacology, Antoni van Leeuwenhoek Hospital/Netherlands Cancer Institute, Amsterdam, the Netherlands; 2 Drugs for Neglected Diseases initiative, Geneva, Switzerland; 3 Institute of Endemic Diseases, University of Khartoum, Khartoum, Sudan; 4 Centre for Clinical Research, Kenya Medical Research Institute, Nairobi, Kenya; 5 Department of Medical Microbiology, College of Health Sciences, Makerere University, Kampala, Uganda; 6 Drugs for Neglected Diseases initiative (DNDi), Nairobi, Kenya; 7 Department of Pharmacy, Uppsala University, Uppsala, Sweden; 8 Department of Clinical Pharmacy, University Medical Center Utrecht, Utrecht University, Utrecht, The Netherlands; 9 Department of Pharmacology, Princess Máxima Center for Pediatric Oncology, Utrecht, The Netherlands; 10 Department of Medical Microbiology and Infection Prevention, Laboratory for Experimental Parasitology, Academic Medical Center, Amsterdam, the Netherlands; Keele University, UNITED KINGDOM

## Abstract

**Background:**

With the current treatment options for visceral leishmaniasis (VL), recrudescence of the parasite is seen in a proportion of patients. Understanding parasite dynamics is crucial to improving treatment efficacy and predicting patient relapse in cases of VL. This study aimed to characterize the kinetics of circulating *Leishmania* parasites in the blood, during and after different antileishmanial therapies, and to find predictors for clinical relapse of disease.

**Methods:**

Data from three clinical trials, in which Eastern African VL patients received various antileishmanial regimens, were combined in this study. *Leishmania* kinetoplast DNA was quantified in whole blood with real-time quantitative PCR (qPCR) before, during, and up to six months after treatment. An integrated population pharmacokinetic-pharmacodynamic model was developed using non-linear mixed effects modelling.

**Results:**

Parasite proliferation was best described by an exponential growth model, with an *in vivo* parasite doubling time of 7.8 days (RSE 12%). Parasite killing by fexinidazole, liposomal amphotericin B, sodium stibogluconate, and miltefosine was best described by linear models directly relating drug concentrations to the parasite elimination rate. After treatment, parasite growth was assumed to be suppressed by the host immune system, described by an E_max_ model driven by the time after treatment. No predictors for the high variability in onset and magnitude of the immune response could be identified. Model-based individual predictions of blood parasite load on Day 28 and Day 56 after start of treatment were predictive for clinical relapse of disease.

**Conclusion:**

This semi-mechanistic pharmacokinetic-pharmacodynamic model adequately captured the blood parasite dynamics during and after treatment, and revealed that high blood parasite loads on Day 28 and Day 56 after start of treatment are an early indication for VL relapse, which could be a useful biomarker to assess treatment efficacy of a treatment regimen in a clinical trial setting.

## Author summary

After primary treatment of visceral leishmaniasis (VL), relapse of disease occurs in a proportion of patients. Understanding parasite dynamics is crucial to improving treatment efficacy and predicting disease relapse at an early stage. *Leishmania* kinetoplast DNA loads in blood from East African VL patients from three clinical trials, treated with five different treatment regimens, were used to develop a semi-mechanistic model to integrate *in vivo* parasite replication in the host, parasite clearance by different VL drug regimens, and suppression of parasite regrowth by the host immune system after treatment. This model deepened our understanding of the parasite-drug-host interaction and described the *in vivo* parasite growth rate in human for the first time. Moreover, the model revealed that high blood parasite loads on Day 28 and Day 56 after start of treatment are an early indication for VL relapse, which could serve as a biomarker to predict long-term clinical outcome, an important new tool for optimizing future VL treatment regimens.

## 1. Introduction

In Eastern Africa, the region with the highest visceral leishmaniasis (VL) incidence globally, efficacy rates of currently available VL therapies range from 72% to 91% [1–3]. Almost all patients show a good initial response to drug treatment with improvement in the clinical signs and symptoms and a negative parasitological test of cure by microscopy at the end of treatment, fulfilling the definition of initial cure. Therapy failure occurs mainly by relapse of disease after initial successful treatment due to parasite recrudescence, which is a long-term event that can occur up to 12 months after treatment, or even longer [4]. Following successful treatment of VL infection, latent parasites may still be present and can be reactivated, resulting in recurrence of VL once immunity is compromised [5–8]. Patients who start VL treatment are mostly malnourished and severely sick, often presenting fever, hypergammaglobulinaemia, and haematological depletions such as anaemia, neutropaenia, and leucopaenia [9]. These complications may lead to an impaired functioning of the immune system, which is unable to suppress or eradicate *Leishmania* parasites [10]. Once the patient improves after the start of treatment, the immune system can recover and clear or control the parasites.

It is particularly difficult to predict which patients will relapse, as almost all patients are clinically cured at the end of treatment. Quantification of the total *Leishmania* parasite burden in patients might give a good approximation of the severity of disease and response to treatment. The gold standard in clinical trials is quantification of parasites by microscopy in aspirates from spleen or bone marrow, where the mainstay of parasites is resident, performed before start of treatment to confirm VL infection, at the end of treatment to assess initial cure, and when relapse is suspected. However, aspiration is a highly invasive procedure and is, therefore, not suitable for more regulator monitoring. Quantification of circulating *Leishmania* kinetoplast DNA (kDNA) in blood by real-time quantitative PCR (qPCR) is an attractive patient-friendly alternative, which allows the collection of longitudinal data. Previously, qPCR parasite load in blood showed a good correlation with qPCR parasite load in aspirated organ tissue (*ρ* = 0.80) [11], indicating that whole blood is an adequate proxy compartment for monitoring parasite biomass in the infected organs. Positive blood parasite load after treatment has been associated with a higher risk of VL relapse [12–24], and the blood parasite load on day 56 after start of treatment has been found to be a highly sensitive predictor of relapse at a cut-off of 20 parasites/mL [11]. This low cut-off value indicates that very low blood parasite loads are already associated with a higher risk of disease relapse, even when patients do not yet present reoccurrence of clinical symptoms.

The phenomenon of asymptomatic parasite recrudescence without clinical relapse complicates the analysis of parasite dynamics in relation to clinical response [11]. Analysis of parasite dynamics is further complicated by the large baseline variability of parasite load and the large inter-patient variability in response [11]. Factors that affect parasite dynamics and treatment response may depend on the initial degree of parasite depletion by the treatment, but also on parasite-related factors such as of the total burden of parasites present at the start of treatment or the virulence of the parasite. Sufficient suppression of the parasite by a properly functioning host immune system is of crucial importance for achieving long-lasting cure; this might be affected by patient-specific factors such as severity of VL infection, co-infections such as HIV, or malnutrition.

A dynamic pharmacokinetic-pharmacodynamic model is needed to capture the interplay between parasite growth, drug exposure, drug-induced parasite clearance, and suppression of parasite regrowth after treatment of the host. While pharmacokinetic-pharmacodynamic models have been shown to be useful for the understanding of other parasitic diseases, such as malaria [25–28], *in vivo* parasite replication rates or parasite clearance by VL drugs have not previously been studied and quantified for *Leishmania*. Longitudinal analysis of repeated blood parasite loads during and after VL treatment will enable characterization of these dynamics [11].

In this study, we aimed to develop a pharmacokinetic-pharmacodynamic model of *Leishmania* blood parasite loads in VL patients receiving various drug regimens, to get a better understanding of the interplay between parasite, drugs, and host. Moreover, using a semi-mechanistic model we characterized the different effects of VL treatment on parasite clearance during treatment, to further enable optimization of dosing regimens or new combination regimens. Lastly, to predict the long-term response of *Leishmania* parasites to treatment, we aimed to identify early biomarkers or model-derived predictors for parasite recrudescence and clinical relapse of disease.

## 2. Methods

### 2.1 Ethics statement

The samples analysed originated from three Phase II open-label randomized clinical trials, that assessed the safety and efficacy of different VL treatment regimens in Eastern Africa (Table 1). Ethical approval was obtained from independent ethics committee at the Faculty of Medicine, University of Khartoum and the Sudanese National Medicines and Poisons Board in Sudan, the London School of Hygiene & Tropical Medicine Ethics Committee (#5543 and #6351), a ’declaration of no objection’ from the Academic Medical Center Medical Ethics Committee (LEAP0208), and from institutional ethics committees at the Kenya Medical Research Institute and at Makerere University, Uganda (LEAP0208, LEAP0714), and the Institute of Endemic Diseases at the University of Khartoum (Fexi-VL-001). Study participants or their parents/guardians (for children under 18 years) provided written informed consent before enrolment into the study, including participation in the pharmacokinetic and parasitological assessments.

**Table 1:** Patient characteristics and data overview.

Study	LEAP0208			LEAP0714	FEXI-VL-001	Total
Treatment	AmB+SSG10D	AmB+MF10D	MFC28D	MFA28D	Fexi10D	
Patients^a^ (*n*)	40	44	46	29	13	172
Cured patients (*n* [%])	37 (93)	37 (84)	35 (76)	27 (93)	2 (15)	138 (80)
Male (*n* [%])	28 (70)	36 (82)	41 (89)	8 (28)	1 (8)	114 (66)
Age (years)^b^	14 (7–40)	14 (7–30)	15 (7–37)	8 (4–12)	26 (16–50)	14 (4–50)
Body weight (kg)^b^	33 (15–69)	34 (15–57)	36 (16–60)	22 (13–30)	51 (42–64)	34 (13–69)
PD samples^c^ collected (n)	306	306	313	208	127	1260
PD samples^c^ included (n [% excluded])	243 (21)	256 (16)	278 (11)	138 (34)	77 (39)	992 (21)

AmB+MF10D: Amphotericin B 10 mg/kg (1 day) + miltefosine 2.5 mg/kg (10 days); AmB+SSG10D: Amphotericin B 10 mg/kg (1 day) + SSG 20 mg/kg (10 days); Fexi10D: Fexinidazole 1800 mg (4 days), 1200 mg (6 days); MFC28D: Miltefosine conventional 2.5 mg/kg (28 days), MFA28D: Miltefosine allometric dosing (28 days)

^a^ Patients included in the analysis

^b^ Mean (range) at baseline

^c^ Samples for quantification of blood parasite load by qPCR

### 2.2 Study design, patients, and clinical assessment of efficacy

In clinical trial LEAP0208 (NCT01067443 [1]) three different treatment regimens in patients from Kenya (Kimalel) and Sudan (Dooka and Kassab) were compared: (1) a combination of 10 mg/kg liposomal amphotericin B (IV) on day 1 followed by 10 days of 20 mg/kg sodium stibogluconate (IM) from day 2–11 (*n* = 51) (AmB+SSG10D), (2) a combination of 10 mg/kg liposomal amphotericin B (AmBisome) (IV) on day 1 followed by 10 days of 2.5 mg/kg/day (maximum 150 mg/day) miltefosine (oral) from day 2–11 (*n* = 49) (AmB+MF10D), (3) monotherapy of conventional miltefosine dosing for 28 days of 2.5 mg/kg/day (maximum 150 mg/day) (*n* = 51) (MFC28D). In clinical trial LEAP0714, (NCT02431143 [2]) 30 paediatric patients from Kenya (Kacheliba) and Uganda (Amudat) who received allometric dosing of miltefosine monotherapy for 28 days (ranging between 30 and 100 mg/day) were studied (MFA28D). In clinical trial FEXI-VL-001 (NCT01980199) a flat dosing of 1800 mg/day fexinidazole for 4 days, followed by 1200 mg/day for 6 days was investigated in 14 adult patients from Sudan (Dooka) (Fexi10D). Patients included in all studies showed VL clinical symptoms (fever and splenomegaly) and had a confirmatory parasitological microscopic diagnosis. All recruited patients were HIV negative. None presented with severe VL, suffered severe malnutrition, or any serious underlying disease or concomitant severe infection at the time of diagnosis.

Clinical assessment of efficacy was defined by initial cure: a negative parasitological test of cure by microscopy on Day 28; final cure: initial cure and absence of VL signs and symptoms until Day 210 (6 months), *i*.*e*., no occurrence of relapse during the follow-up period and no requirement for rescue treatment; or initial treatment failure: patients who died or required rescue treatment before completion of study treatment.

### 2.3 Sample collection, bioanalysis, and data exclusion

Pharmacokinetic samples were collected for miltefosine (LEAP0208 and LEAP0714) and fexinidazole and its active metabolites fexinidazole sulfoxide (M1) and fexinidazole sulfone (M2) (FEXI-VL-001). Miltefosine sample collection was performed in all patients receiving miltefosine monotherapy or a combination therapy with miltefosine. Sample collection and bioanalysis has been described previously [1,29,30]. In FEXI-VL-001, dried-blood spot (DBS) samples were collected at multiple time points during treatment in all patients receiving fexinidazole, with more dense sampling on Day 1 and Day 10 of treatment. Whole blood concentrations of fexinidazole, M1, and M2 were quantified, as described in published literature [31]. Whole blood EDTA samples with a volume of 200 μL were collected for pharmacodynamic assessment in all patients prior to treatment and on Days 3, 7, 14, 28, 56, and 210 (LEAP0208), on Days 3, 7, 14, 21, 28, and 56 (LEAP0714), and on Days 1, 3, 5, 8, 11, 14, 28, 56, and 210 (FEXI-VL-001). *Leishmania* kinetoplastid DNA (kDNA) was quantified in these samples using a qPCR method, to determine the parasite load in the patient (hereafter referred to as blood parasite load). Human glyceraldehyde 3-phosphate dehydrogenase (GAPDH) DNA was used as an internal control for validation of the extraction procedure. The lower limit of quantification (LLOQ) was set to 1 parasite/mL. A detailed description of the DNA extraction, primers used, and qPCR protocol has been described earlier [11].

qPCR data were excluded from the analysis for patients who were considered initial treatment failures and did not complete the study treatment, and for samples collected after rescue treatment was given to relapsed patients, and for samples considered to be unreliable (*i*.*e*., if the GAPDH response indicated unreliable or incomplete DNA extraction of the sample, if the sample quality was low, or if there was insufficient sample material). In addition, qPCR data were excluded if values were physiologically implausible, *i*.*e*. if baseline parasite loads were exceedingly low (<100 parasites/mL), while later parasite loads in the same individual in the first week of treatment indicated much higher loads. Furthermore, patients who received miltefosine or fexinidazole for whom no pharmacokinetic data were available were also excluded.

### 2.4 Population pharmacokinetic-pharmacodynamic analysis

An integrated pharmacokinetic-pharmacodynamic model was developed using the nonlinear mixed effects modelling software NONMEM (version 7.5, ICON Development Solutions, USA) using the first-order conditional estimation method with interaction (FOCE-I). Data cleaning and visualization of the data were performed using R (version 4.0.2), and model evaluation was done using Perl-speaks-NONMEM (PsN, version 5.2.6) and the interface Pirana (version 3.0.0). Model development was performed in three consecutive steps: 1) characterization of parasite growth, 2) characterization of drug-induced parasite clearance during treatment, and 3) characterization of host-induced parasite suppression after treatment. In each step, a relevant subset of the data was used to estimate the different model parameters. Blood parasite loads below the limit of quantification (BLQ) were fixed to half the BLQ (0.5 parasite/mL). Between-subject variability (BSV) was evaluated for all parameters and implemented using an exponential error model. Residual variability was modeled using combined additive and proportional error models, with the additive error fixed to BLQ/2 (0.5 parasite/mL).

#### 2.4.1 Parasite growth

No pharmacodynamic data was available before start of treatment, complicating characterization of natural parasite growth in primary VL patients. *In vivo* parasite growth was, therefore, modelled and estimated based on data from patients treated with fexinidazole, where an inadequate drug response that resulted in rapid parasite recrudescence directly after treatment was observed in the majority of patients. To describe parasite proliferation, turn-over and exponential growth models were evaluated.

#### 2.4.2 Drug-induced parasite clearance during treatment

Previously developed population pharmacokinetic models of miltefosine [29] and fexinidazole and its active metabolites M1 and M2 (internal report) were used to derive individual predicted pharmacokinetic concentrations, which were used as pharmacokinetic input to the model. For liposomal amphotericin B and SSG, a kinetic-pharmacodynamic approach was used assuming a one compartment model with first-order elimination, using the administered drug amounts and previously reported elimination half-lives of 6 hours for liposomal amphotericin B [32] and 2 hours for SSG [33,34]. Direct and delayed sigmoidal E_max_ and linear models were evaluated to model drug-induced killing of parasites driven by individual predicted concentration-time curves for each drug.

#### 2.4.3 Host-induced parasite suppression after treatment

Visual inspection of individual blood parasite loads over time indicated diverse and highly variable profiles after treatment (Fig A in S1 File). Typical profiles after treatment included 1) complete parasite clearance with no parasite recrudescence during follow-up (complete parasitological cure) 2) initial parasite clearance followed by parasite recrudescence, where parasite regrowth is initiated at different time points during the follow-up period, and 3) initial parasite clearance followed by parasite recrudescence early after treatment, followed by parasite clearance later during follow-up (Table A in S1 File).

The aim of this part of building the model was to enable description of these three distinct typical parasite load-time profiles during the follow-up phase after treatment. Based on the hypothesis that parasite suppression after treatment, in the absence of drug pressure, is driven by the host immune system, the variable onset in parasite suppression and clearance after the end of treatment by the host immune system was implemented by a sigmoidal E_max_ function, driven empirically by the time after treatment, which captured differences in onset and magnitude of parasite recrudescence among patients (Eq 1). To allow for clinically meaningful parasite recrudescence after complete drug-induced parasite depletion, the parasite compartment was restricted to ≥1 parasite/mL.


$$k_{imm}=\frac{I_{max}*{Time}^{\gamma }}{{{IT}_{50}}^{\gamma }+{Time}^{\gamma }}$$ Eq 1

In Eq 1, k_imm_ is parasite suppression by the immune system, Time is the time after start of treatment, I_max_ is the maximum inhibitory effect of the immune system, and IT_50_ is the time after treatment of half the maximal immune effect. The parameter γ denotes the steepness of the time-effect relationship.

With the aim of finding biomarkers predictive of (suppression of) parasite recrudescence after therapy, haematological and biochemical markers were evaluated as drivers of parasite suppression (k_imm_) rather than time after treatment. Available haematological and biochemical data included haemoglobin, white blood cells, platelets, and creatinine for all patients, neutrophils, lymphocytes, and monocytes (LEAP0714 and FEXI-VL-001), and total protein and albumin (FEXI-VL-001). All these covariates were evaluated graphically to assess their relationship with parasite recrudescence and suppression. White blood cell count was a physiologically plausible covariate as it may reflect the overall activity of the immune system. Therefore, white blood cell counts on Day 56, either absolute or relative to baseline, was evaluated on parasite suppression by two strategies. First, white blood cell count was evaluated as covariate on the extent of suppression of parasite regrowth after treatment (Eq 1, I_max_) and the onset of parasite suppression after treatment (Eq 1, IT_50_) using a slope function (Eq 2).

$$P_{TV}=P_{pop}*(1+{(WBC}_{i}-{WBC}_{med})*l)$$ Eq 2

where P_TV_ is the typical parameter value for IT_50_ or I_max_ at covariate value WBC_i_, P_pop_ the population estimate of IT_50_ or I_max_, WBC_i_ the white blood cell count or the white blood cell count relative to baseline for individual i on Day 56, WBC_med_ the median covariate value in the population, and *l* the slope factor.

Second, white blood cell count was used as a descriptor for assessing parasite suppression, instead of using empirical time after treatment (Eq 3).


$$k_{imm}=\frac{I_{max}*{{WBC}_{i}}^{\gamma }}{{{IC}_{50}}^{\gamma }+{{WBC}_{i}}^{\gamma }}$$ Eq 3

In Eq 3, k_imm_ is parasite suppression by the immune system, WBC_i_ the white blood cell count or the white blood cell count relative to baseline for individual i on Day 56, I_max_ is the maximum inhibitory effect of the immune system, and IC_50_ is the white blood cell count of half the maximal immune effect. The parameter γ denotes the steepness of the time-effect relationship.

### 2.5 Model evaluation

The aim of this modeling analysis was to describe parasite clearance rates for the different treatment regimens, as well as to capture the different parasite profiles after treatment, which are driven by the immune system. Standard goodness of fit plots and individual prediction plots were used to guide model development. Model selection was based on scientific plausibility and minimum objective function value (OFV), where a decrease of 6.63 points in OFV corresponding to a P value <0.01 was considered significant, with 1 degree of freedom following a χ^2^ distribution. Precision in parameter estimates was obtained by sampling importance resampling (SIR). To further assess the goodness of fit and to compare the model description of parasite clearance during the treatment period in more detail, a visual predictive check of blood parasite loads during treatment was performed for all treatment regimens, in which the effects of both drugs’ PK, parasite growth and the immune response are included. Simulations of parasite profiles were performed in a typical patient receiving different treatment regimens, and having different immune system activities, to illustrate the effect of different drugs and different immune effects.

### 2.6 Evaluation of the correlation between parasite exposure and clinical outcome

One of the goals of the model was to evaluate the predictiveness of parasite load or parasite clearance for subsequent clinical relapse of VL during follow-up. To characterize the relationship of early drug-induced parasite clearance and long-term clinical outcome, different parasite exposure metrics during and early after treatment were evaluated. The sensitivity and specificity of predicting clinical relapse based on blood parasite load on Day 10, Day 28, and Day 56 and the optimal cutoff values were derived by receiver-operating characteristic (ROC) curves, generated using the R packages “pROC” and “plotROC”. The area under the ROC curves (AUC_ROC_) were compared to find the most predictive parameter for clinical relapse in terms of follow-up day (Day 10, Day 28, or Day 56). The fraction of patients having a blood parasite load below the optimal cutoff value resulting from the ROC curve was compared between treatment regimens and between cured and relapsed patients. Second, model-based predictions of parasite load and parasite exposure during and early after treatment (at baseline and Day 10, 28, and 56) were compared between treatment regimens and to clinical outcome. Cumulative exposure to parasites during and after treatment, expressed as the area under the blood parasite load-time curve (AUC), was also compared as a measure of parasite clearance during therapy. This parameter incorporates both the drug-induced parasite clearance rate and the baseline parasite load, which was highly variable among patients. The parasite AUC during the first 10, 28, and 56 days were compared. I_max_ and IT_50_ were compared to assess any treatment-related effects on the suppression of parasite recrudescence after treatment.

## Results

### 3.1 Data

**Fig 1:**
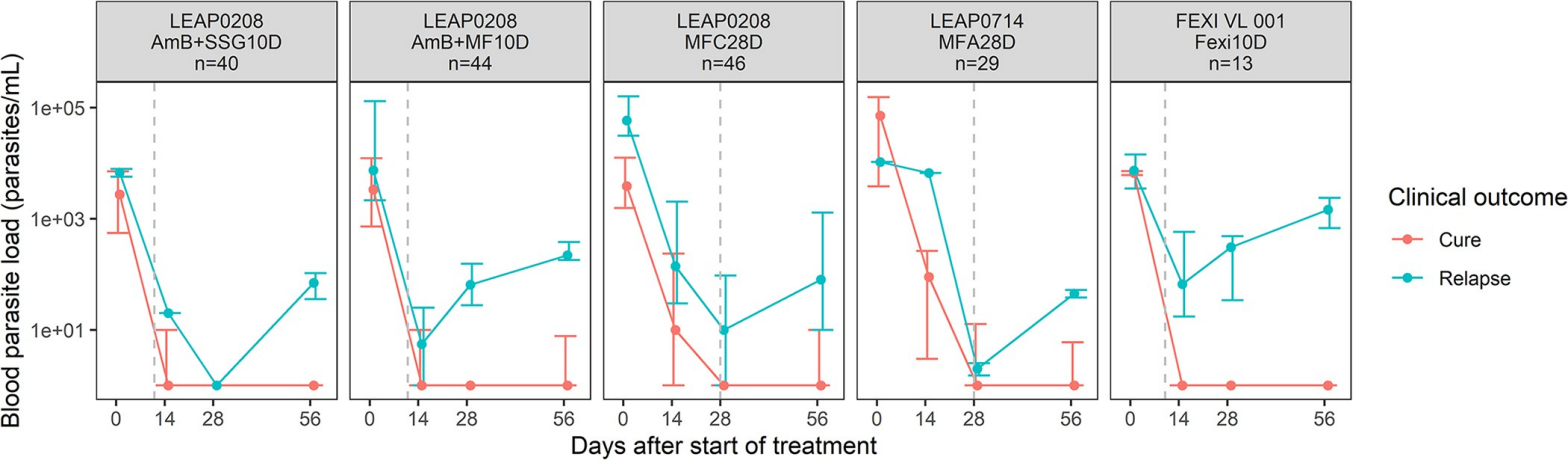
Blood parasite loads in Eastern African visceral leishmaniasis patients during treatment and early follow-up. Depicted are median (IQR) observed blood parasite loads coloured by treatment outcome (cured patients in red and relapsed patients in blue), stratified by treatment regimen. AmB+SSG10D: 10 mg/kg amphotericin B (day 1) + 20 mg/kg/day SSG (day 2 to 11); AmB+MF10D: 10 mg/kg amphotericin B (day 1) + 100 mg/day miltefosine (day 2 to 11); MFC28D: miltefosine conventional dose (100 mg/day) (28 days); MFA28D: miltefosine allometric dose (28 days); Fexi10D: 1800 mg/day fexinidazole (4 days) + 1200 mg/day fexinidazole (6 days). Gray dashed lines represent the end of treatment.

In total, 1260 blood samples from 188 patients were available for qPCR analysis (Table 1). The following qPCR observations were excluded from the analysis: 2 initial treatment failures (7 observations), 21 observations collected after rescue treatment, 190 unreliably extracted samples, and 44 physiologically impossible observations (3.5%). One patient who received AmB+MF10D but lacking pharmacokinetic data was excluded (6 observations). Of the 992 blood parasite load observations included in the analysis, 359 observations (36%) were BLQ. An overview of the data included in the analysis up to Day 56 is shown in Fig 1, which displays trends in the data during and early after treatment between the different regimens.

### 3.2 Population pharmacokinetic-pharmacodynamic model

#### 3.2.1 Parasite growth and drug-dependent parasite clearance

**Fig 2:**
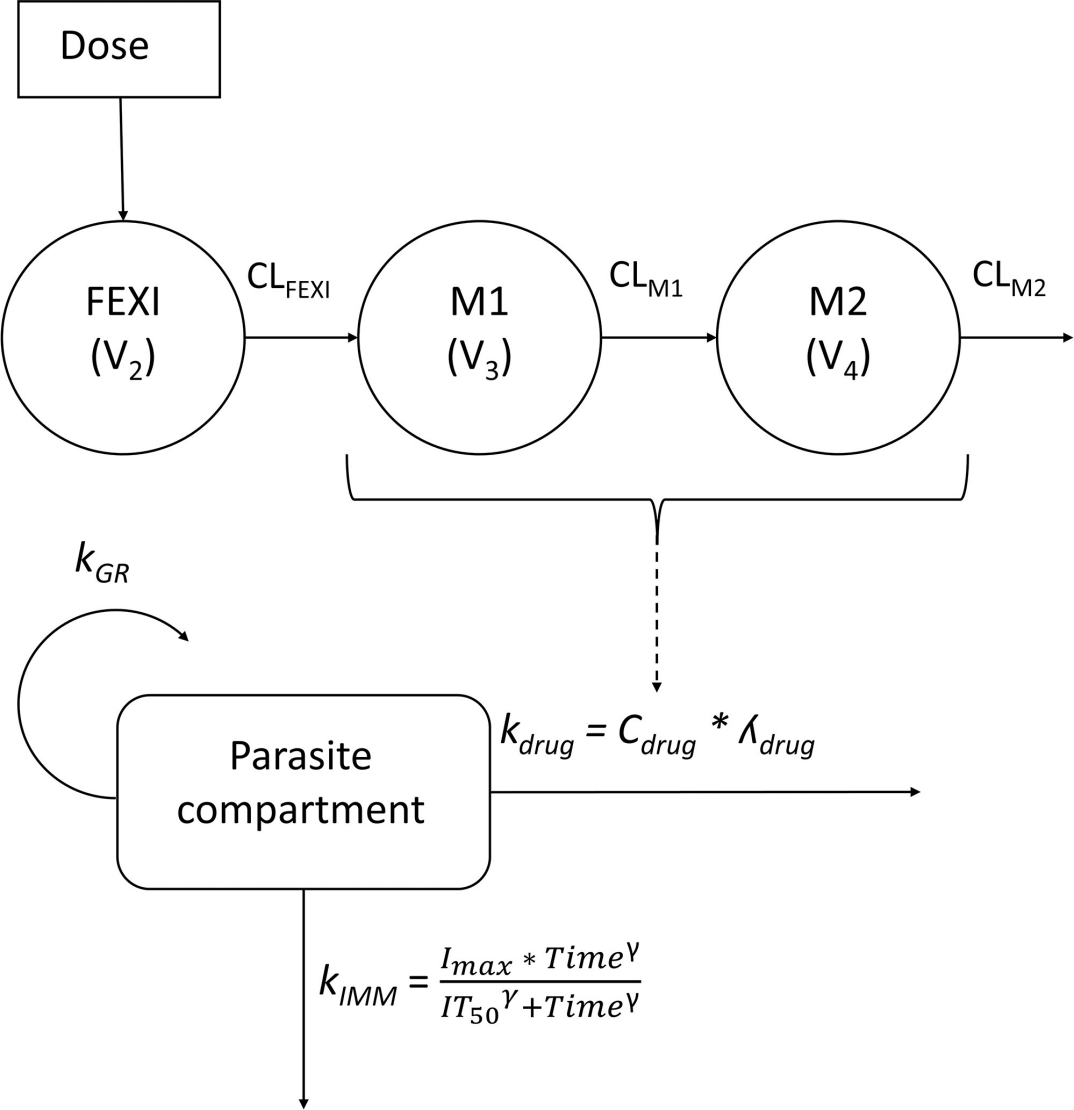
Schematic overview of the final pharmacokinetic-pharmacodynamic model, exemplified with the pharmacokinetic model for fexinidazole and its active metabolites M1 and M2. In the parasite model, k_GR_ is the parasite replication rate, k_drug_ is the drug-driven parasite clearance rate, λ_drug_ the drug-specific linear effect, and C_drug_ the drug concentration of either miltefosine, the sum of M1 and M2 for fexinidazole, amphotericin B, or SSG. k_IMM_ is the immune-driven parasite clearance, I_max_ the maximum inhibition by the immune response, IT_50_ the time at half-maximum inhibition, and γ the steepness of the time-effect relationship, which was empirically fixed to 5. In the fexinidazole pharmacokinetic model, CL is the clearance of fexinidazole, M1 or M2, and V_2_, V_3_, and V_4_ the volume of distribution of fexinidazole, M1, and M2, respectively.

**Fig 3:**
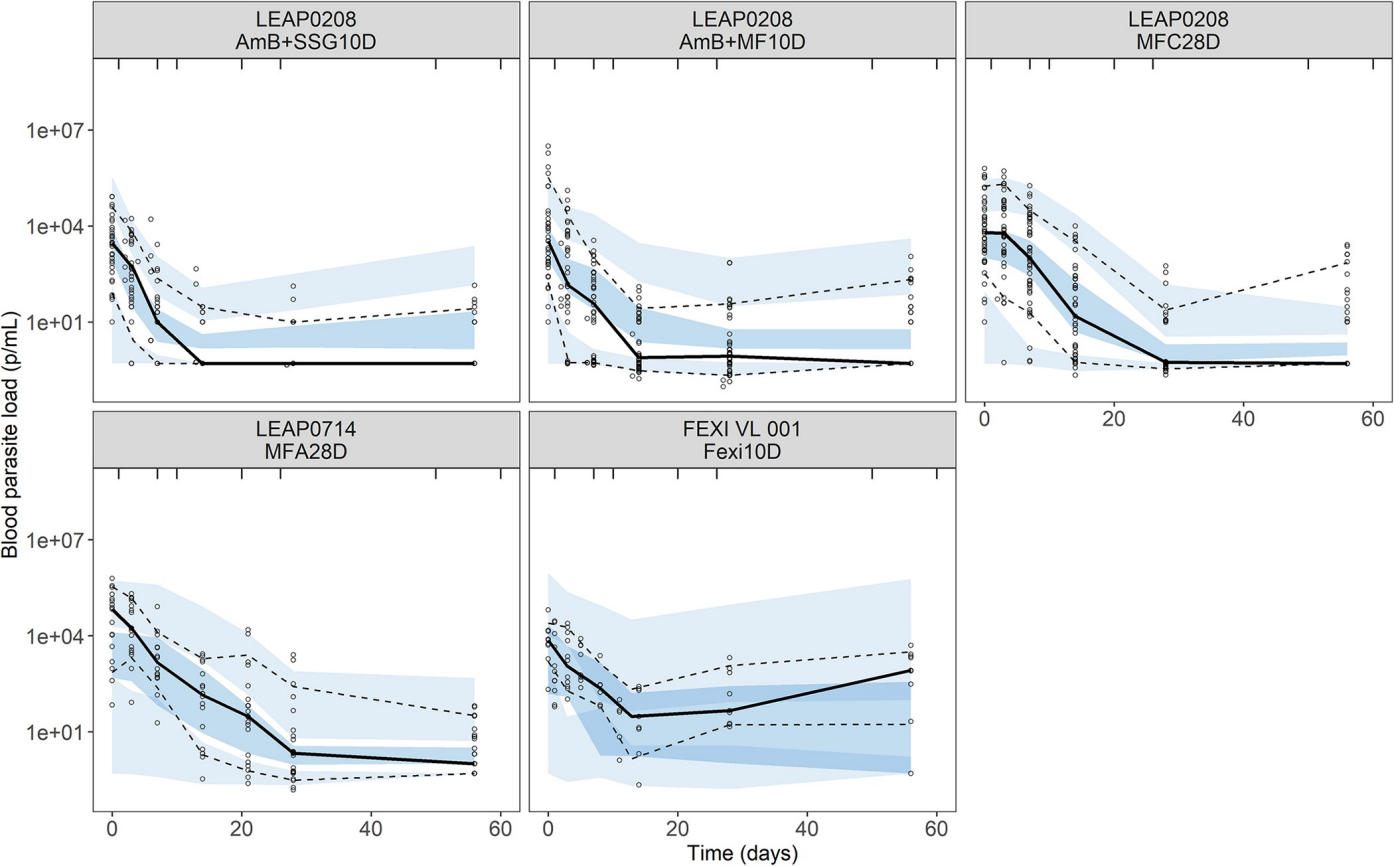
Prediction-corrected visual predictive checks for the final pharmacokinetic-pharmacodynamic blood *Leishmania* parasite load model until Day 56 after start of treatment. Solid lines: median of the observed values; dashed lines: the 10^th^ and 90^th^ percentiles of the observed values; dark and light blue areas: the 90% confidence intervals of the simulated median and percentiles, based on 1000 simulations.

**Fig 4:**
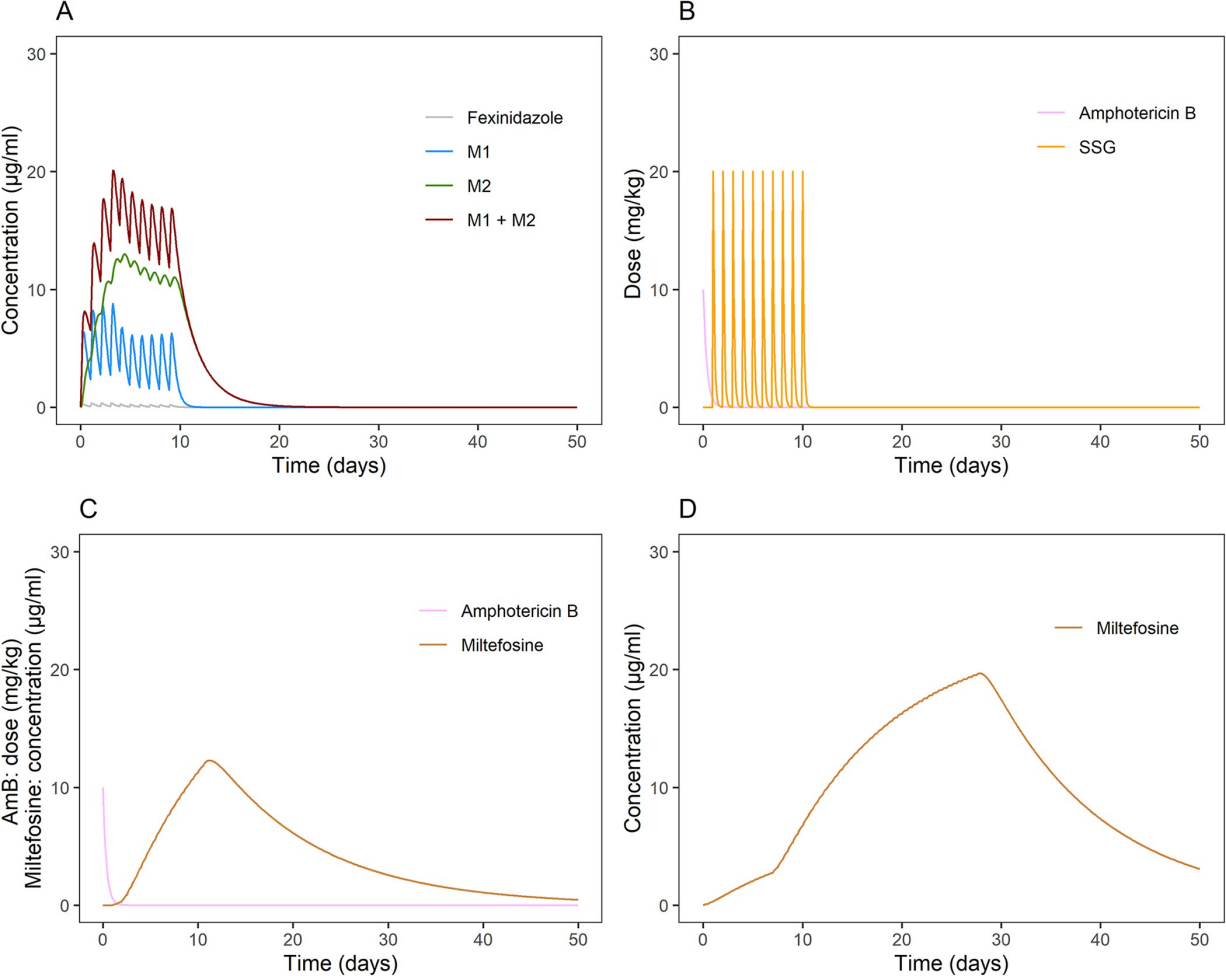
Simulations of typical pharmacokinetic profiles of patients receiving A) 1800 mg/day fexinidazole (4 days) + 1200 mg/day fexinidazole; B) 10 mg/kg amphotericin B (day 1) + 20 mg/kg/day SSG (day 2 to 11); C) 10 mg/kg amphotericin B (day 1) + 100 mg/day miltefosine (day 2 to 11); or D) 100 mg/day miltefosine (28 days).

**Fig 5:**
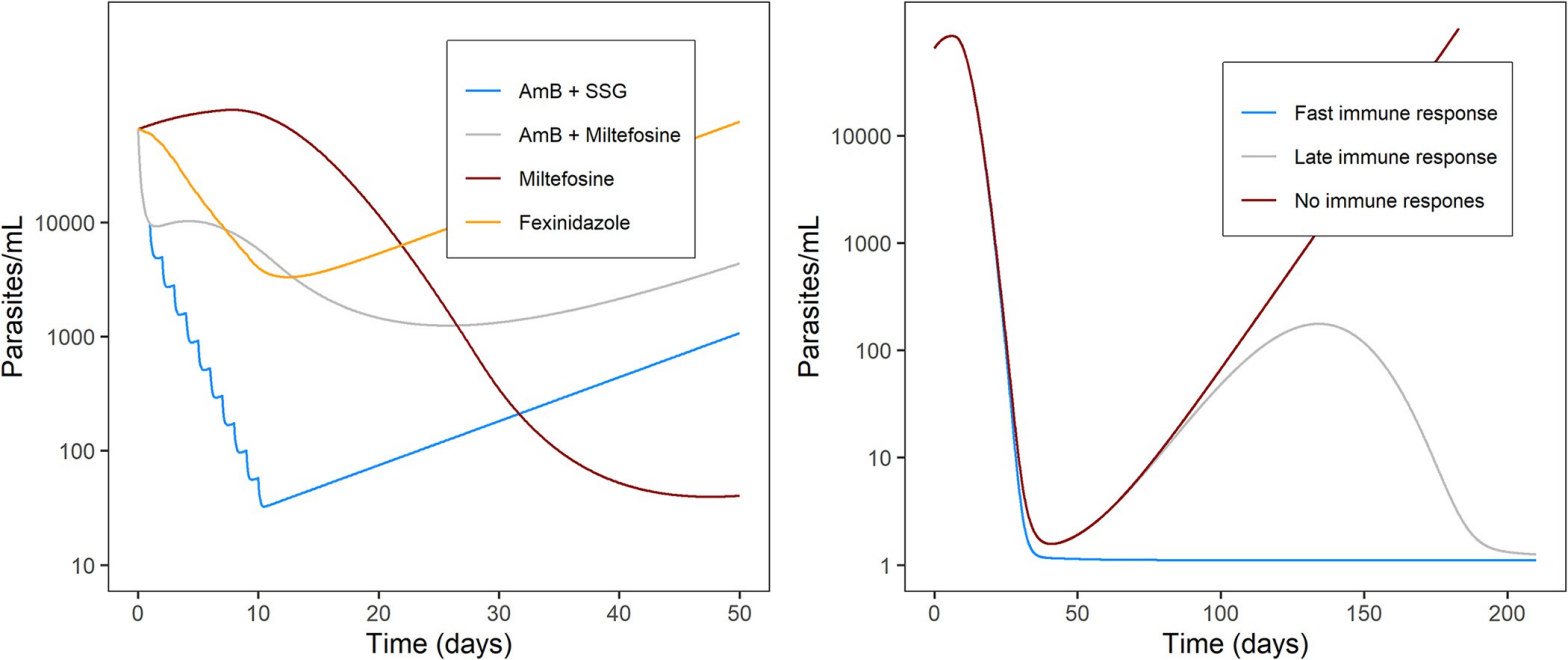
A (left plot): Simulation of drug effects of different VL therapies of typical patients receiving 1) 10 mg/kg amphotericin B (day 1) + 20 mg/kg/day SSG (day 2 to 11) (blue curve), 2) 10 mg/kg amphotericin B (day 1) + 100 mg/day miltefosine (day 2 to 11) (grey curve), 3) 100 mg/day miltefosine (28 days) (red curve), or 4) 1800 mg/day fexinidazole (4 days) + 1200 mg/day fexinidazole (6 days) (yellow curve). No immune response after the end of treatment was observed. Other parameters were fixed to the population values. B (right plot): Simulation of typical patients receiving 150 mg/day miltefosine for 28 days. Patients have an IT_50_ of 1000 h (blue curve), 5000 h (grey curve), and 100,000 h (red curve). Other parameters were fixed to the population values.

Parasite proliferation was best described by an exponential growth model (Fig 2), with an *in vivo* parasite doubling time of 7.8 days (RSE 12%) (Table 2 and Fig 2). The model-derived individual predictions for the Fexi10D regimen indicated that parasite proliferation was adequately described, as the model captured parasite growth after treatment in these patients (Fig A in S1 File). Drug-dependent parasite killing was best described by first-order linear pharmacokinetic-pharmacodynamic models (fexinidazole and miltefosine) or kinetic-pharmacodynamic models (liposomal amphotericin B and SSG), where the parasite killing rate was directly proportional to the drug concentration. The visual predictive check shows that the model quite adequately described parasite clearance in the majority of treatment regimens (Fig 3). There were some discrepancies between the observed data and the model simulations, i.e., parasite recrudescence in Fexi10D was under-predicted by the model, baseline blood parasite load in MFA28D was under-predicted, and the parasite clearance rate in AmB+MF10D was under-predicted, especially the upper boundary of the prediction interval. The time course of drug exposure in the different treatment regimens was simulated for a typical patient (Fig 4), illustrating variable durations of exposure depending on the pharmacokinetic characteristics of the drugs and their corresponding elimination half-lives, which were much longer for miltefosine. This resulted in persistent drug exposure of about 11 days for the AmB+SSG10D and Fexi10D regimens, 30 days for the AmB+MF10D regimen, and 50 days for the MFC28D and MFA28D regimens. The individual model-based predictions during treatment further illustrate different parasite clearance rates for the various drug regimens (Fig A and Fig B in S1 File). The differential pharmacokinetic profiles, in combination with the drug-specific drug effects on parasite clearance, led to different patterns of parasite dynamics during the treatment period for each treatment regimen (Fig 5A). Rapid and effective parasite clearance was induced by treatment with liposomal amphotericin B (AmB+SSG10D and AmB+MF10D), while a slow onset with later parasite clearance was observed for miltefosine (MFC28D and MFA28D), and a weak response was observed for fexinidazole (Fexi10D).

**Table 2:** Parameter estimates for the final population pharmacokinetic-pharmacodynamic model.

Parameter	Parameter estimate	RSE (%)^a^	BSV (CV%)	RSE (%)^a^	Shrinkage (%)
E_BASE_ (parasites/mL)	5324	16	243	12	3
k_GR_ (h^-1^)	0.0037	12	-	-	-
λ_fexi_ (μg^-1^*L*h^-1^)	0.0011	15	44	59	3
λ_MF_ (μg^-1^*L*h^-1^)	0.0010	5	42	21	5
λ_Amb_ (mg^-1^*kg*h^-1^)	0.0245	8	-	-	-
λ_SSG_ (mg^-1^*kg*h^-1^)	0.0112	5	-	-	-
I_max_ (h^-1^)	0.037 (fixed)^b^	-	298	16	52
IT_50_ (days)	33.7	0.1	230	12	40
Proportional residual error (%)	101	-		0.7	18
Additive residual error (parasites/mL)	0.5 (fixed)^c^	-		-	18

BSV: between-subject variability; CV: coefficient of variation; E_BASE_: Baseline blood parasite load; I_max_: maximum inhibition by immune response; IT_50_: time at half-maximum inhibition; k_GR_: parasite growth constant; λ_Amb_: linear drug effect Amphotericin B; λ_fexi_: linear drug effect fexinidazole; λ_MF_: linear drug effect miltefosine; λ_SSG_: linear drug effect SSG; RSE: relative standard error

^a^ Obtained by SIR

^b^ Fixed to ten times k_GR_

^c^ Fixed to half the lower limit of quantification

#### 3.2.2 Parasite suppression after treatment

Parasite suppression after treatment was empirically described by a first-order elimination process with a sigmoid E_max_ function driven by the time after treatment, with γ empirically fixed to 5, representing a fast onset of parasite suppression by the host’s immune system after start of treatment (Fig 2). The model captured the different parasite profiles after treatment, including complete parasite suppression, parasite regrowth at different time points during follow-up, and initial parasite regrowth followed by later suppression, and could therefore describe most of the individual profiles of parasite recrudescence during follow-up (Fig A and Fig C in S1 File). High variability in recrudescence profiles was reflected in a very large and non-normally distributed BSV (>200 CV%) for baseline parasite load, I_max_, and IT_50_. Typical value plots, as depicted in Fig 5B, illustrate the effect of different onset times of immune suppression (different IT_50_) on the parasite dynamics during follow-up. None of the haematological markers correlated with parasitological response (Fig D in S1 File) and white blood cell counts were not a significant covariate for k_imm_ or IT_50_. Profiles with complete parasite clearance during treatment, followed by a fast and strong parasite recrudescence, could not be fully captured by the model (Fig A in S1 File), despite the restriction of low parasite loads to ≥1 parasites/mL in the model.

### 3.3 Correlation between parasite exposure and clinical outcome

**Fig 6:**
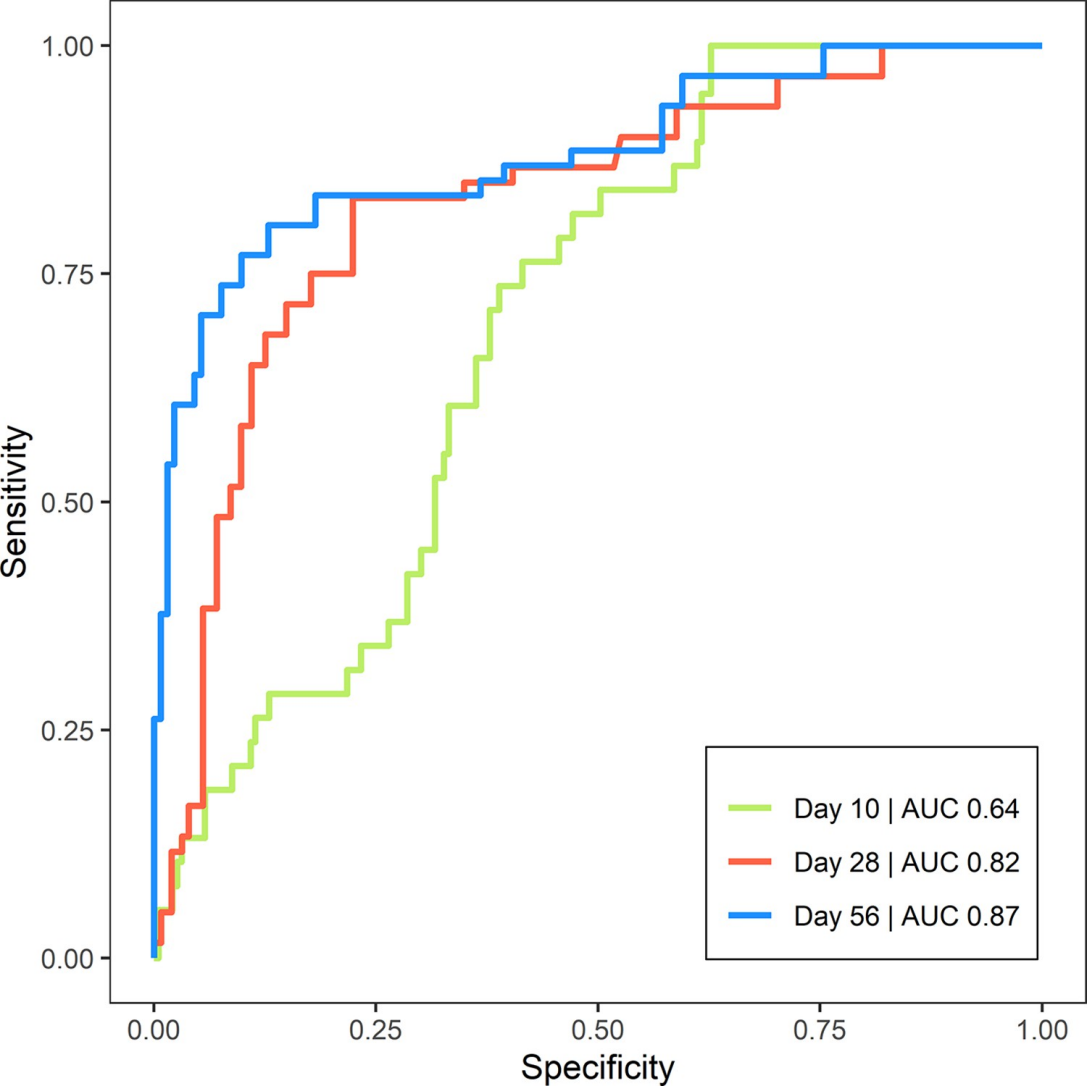
ROC curves of blood parasite load as predictor of clinical relapse on Day 10, 28, and 56 after start of treatment. AUC represents the integrated area under the ROC curve. Green line: day 10 (AUC 0.64), red line: day 28 (AUC 0.82), blue line: day 56 (AUC 0.87). Abbreviations: AUC, area under the curve; ROC, receiver operating characteristic.

The AUC_ROC_ for blood parasite load classifying clinical relapse (Fig 6) was highest on Day 28 (0.82) and Day 56 (0.87), compared with Day 10 (0.64) (Table 3). The optimal cutoff values for classifying relapse were around 10 parasites/mL (9 parasites/mL on Day 28 and 11 parasites/mL on Day 56). The fraction of patients having a blood parasite load <10 parasites/mL on Day 28 and Day 56 was therefore compared between treatment regimens and the final clinical outcome (Table 4). There were considerably more patients (73–86%) with an adequate parasite clearance (parasite load <10 parasites/mL) on Day 56 in relatively effective treatment regimens (AmB+SSG10D, AmB+MF10D, MFC28D, MFA28D), compared to only 10% in the non-efficacious Fexi10D regimen. Based on the cutoff value of 10 parasites/mL, 74% and 76% of relapsed patients were correctly categorized as relapse on Day 28 and Day 56, respectively.

**Table 3:** Performance of blood parasite load on different days after treatment as a predictor for clinical relapse, based on ROC analysis.

Day^a^	AUC_ROC_^b^	Cutoff value (parasites/mL)^c^	Sensitivity	Specificity
10	0.64	51	0.44	0.85
28	0.82	9	0.77	0.82
56	0.87	11	0.88	0.77

^a^ Day after start of treatment

^b^ Area under the ROC curve

^c^ Optimal cutoff value of blood parasite load to predict clinical relapse

**Table 4:** Individual model-based predictions of parasite exposure per treatment arm and clinical outcome.

Study	LEAP0208			LEAP0714	FEXI-VL-001		
Treatment/outcome	AmB+SSG10D	AmB+MF10D	MFC28D	MFA28D	Fexi10D	Cure	Relapse
Patients^a^ (*n*)	40	44	46	29	13	138	34
Cured patients (*n* [%])	37 (93)	37 (84)	35 (76)	27 (93)	2 (15)	138 (100)	0 (0)
Parasite load <10p/mL at D28 (n [%])	31 (78)	31 (70)	32 (70)	18 (62)	3 (10)	106 (77)	9 (26)
Parasite load <10p/mL at D56 (n [%])	33 (83)	32 (73)	34 (74)	25 (86)	3 (10)	119 (86)	8 (24)
Parasite load D1^b^ (p/mL)	23802 (4904–51372)	4493 (553–34633)	4583 (1008–29194)	33363 (6091–77096)	3732 (2605–14467)	7142 (1231–40157)	14467 (4493–62465)
Parasite load D10^b^ (p/mL)	11.8 (2.8–35.9)	137 (4.0–807)	2027 (226–12355))	2378 (429–44318)	105 (25.1–249)	150 (8.8–2094)	333 (122–5985)
Parasite load D28^b^ (p/mL)	1.1 (1.0–6.1)	2.1 (1.0–22.9)	1.1 (1.0–135)	1.3 (1.1–42.2)	112 (18.2–216)	1.1 (1.0–6.1)	105 (11.6–313)
Parasite load D56^b^ (p/mL)	1.0 (1.0–5.7)	1.1 (1.0–10.1)	1.3 (1.0–10.4)	1.2 (1.1–3.1)	486 (11.1–1073)	1.1 (1.0–2.7)	125 (11.9–613)
Parasite AUC_D0-10_^b^ (p·day/mL)	7284 (2385–19935)	5970 (641–41824)	32509 (8631–162007)	39558 (7638–417250)	8482 (4827–36350)	15648 (3241–52869)	17209 (5012–97125)
Parasite AUC_D0-28_^b^ (p·day/mL)	7371 (2537–20370)	7061 (804–47564)	56327 (18322–223201)	74829 (14150–604792)	10260 (5644–37750)	18642 (3382–80241)	24596 (7042–129150)
Parasite AUC_D0-56_^b^ (p·day/mL)	7400 (3094–20972)	10905 (906–52583)	56906 (18973–223236)	75175 (15254–604833)	33768 (8532–69033)	18884 (3464–85774)	41931 (14171–173361)
I_max_ (day^-1^)^c^	2.3 (1.7)	1.9 (2.0)	1.4 (1.6)	1.2 (0.7)	0.9 (0.7)	1.9 (1.7)	0.7 (0.5)
IT_50_ (day)^c^	23 (21)	45 (58)	59 (73)	60 (50)	77 (57)	40 (51)	84 (65)

AmB+MF10D: Amphotericin B 10 mg/kg (1 day) + miltefosine 2.5 mg/kg (10 days); AmB+SSG10D: Amphotericin B 10 mg/kg (1 day) + SSG 20 mg/kg (10 days); BLQ: below the limit of quantification; Fexi10D: Fexinidazole 1800 mg (4 days), 1200 mg (6 days); I_max_: maximum inhibitory effect of the immune system; IT_50_: time after treatment of half the maximal immune effect; MFA28D: miltefosine allometric dosing (pediatrics); MFC28D: Miltefosine conventional dosing (2.5 mg/kg) (adults) p/mL: parasites/mL.

^a^ Patients included in the analysis

^b^ Median (IQR)

^c^ Mean (sd). I_max_ and IT_50_ represent the activity of the immune system in suppressing parasites

**Fig 7:**
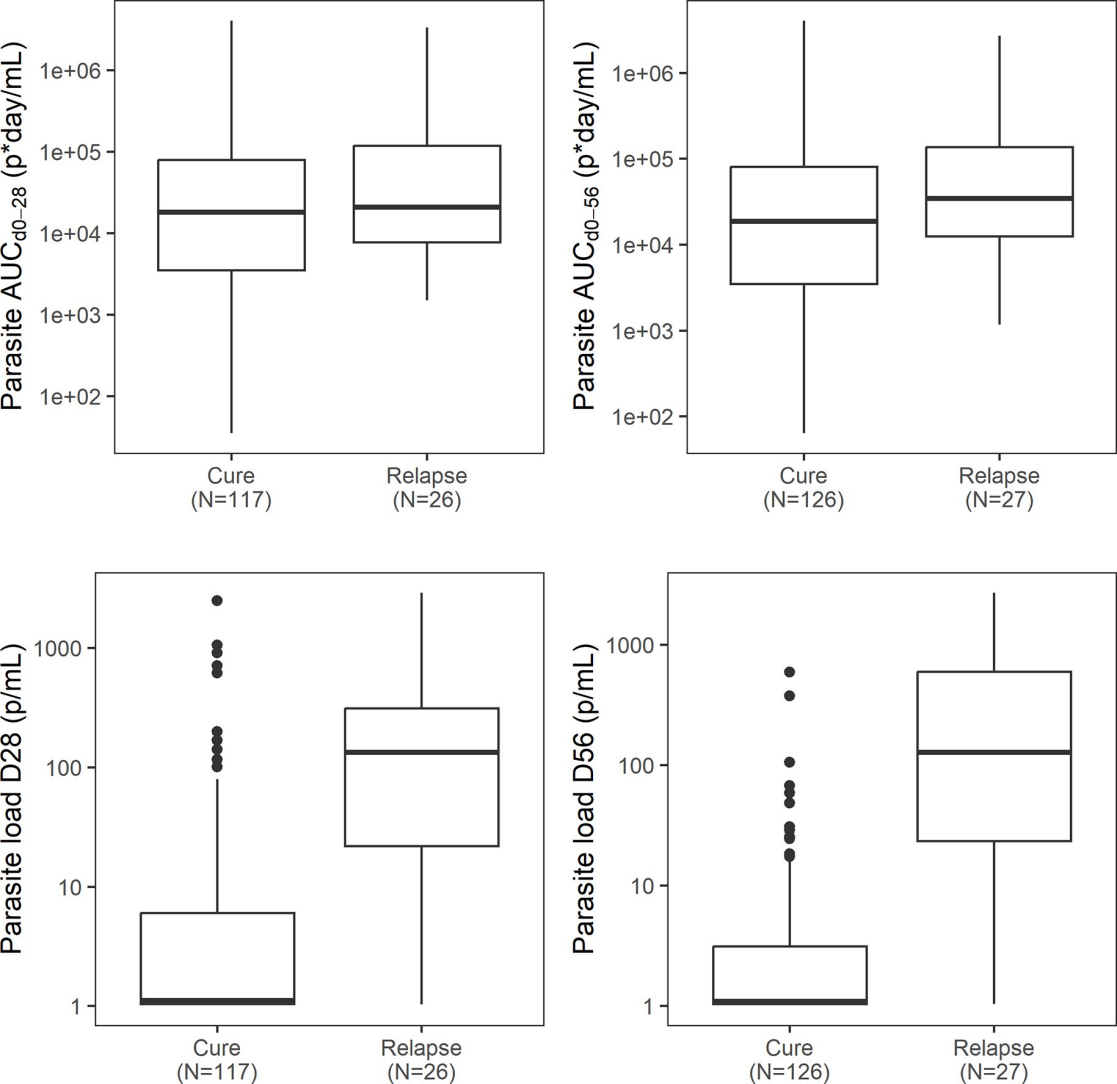
Parasite AUC_d0-28_ and AUC_d0-56_ and parasite load on Day 28 and Day 56 versus clinical outcome.

Various other individual model-based secondary blood parasite load metrics were compared between treatment regimens and the final clinical outcome (Table 4). In the miltefosine monotherapy regimens (MFC28D and MFA28D), the individual predicted blood parasite loads were higher on Day 10 compared to other regimens, but reached and remained low blood parasite loads on Day 28 and Day 56 suggesting high overall efficacy. In the Fexi10D treatment arm, individual predicted blood parasite loads were higher on Day 28 and Day 56, which had poor efficacy. Blood parasite loads on Day 10, Day 28, and Day 56 were substantially higher in relapsed patients (Table 4 and Fig 7).

Although a low parasite AUC for different durations (AUC_D0-10_, AUC_D0-28_, AUC_D0-56_) was not associated with favorable clinical outcome (also not when stratified by treatment regimen), a slightly lower AUC_D0-56_ of 18884 (3464–85774) p·day/mL was observed in cured patients compared to 41931 (14171–173361) p·day/mL in relapsed patients. The parasite AUCs were considerably higher in the miltefosine monotherapy regimens (MFC28D and MFA28D), which is in accordance with the slow miltefosine drug accumulation and slow onset of miltefosine-induced parasite clearance. The extent of suppression of parasite regrowth after treatment (I_max_) did not clearly correlate with treatment efficacy, but the onset of the suppressive immune response (IT_50_) was substantially delayed in the Fexi10D treatment arm, which had poor efficacy, indicating that a weak drug effect and partial parasite clearance during treatment negatively influenced the onset of the immune response. This was in accordance with the delayed IT_50_ in relapsed patients compared to cured patients.

## 4. Discussion

A semi-mechanistic pharmacokinetic-pharmacodynamic model was developed, based on pharmacokinetic and blood parasite load data from Eastern African VL patients receiving five different (combination or monotherapy) treatment regimens, to characterize the complicated interaction between parasite replication, drug-induced parasite clearance, and parasite clearance due to an emerging host immune response. The model adequately captured the blood parasite dynamics during and after treatment and revealed that blood parasite loads higher than 10 parasites/mL on Day 28 and Day 56 after start of treatment are an early indication of VL relapse, which could serve as a biomarker to predict long-term clinical outcome based on the sensitivity and specificity. Until now, it has not been possible to predict relapse, a long-term event that can occur up to 12 months or even longer after treatment. Moreover, the model indicated that the long-term clinical outcome depends both on the initial parasite clearance by the treatment and on parasite suppression after treatment, which is probably achieved by the host immunological response. A better understanding of this immunological response and its associated host biomarkers could potentially lead to an improved prediction of relapse.

Parasite replication and drug-induced parasite clearance by five different VL drug regimens were adequately characterized by the pharmacokinetic-pharmacodynamic model we developed. To our knowledge, the model provided the first-ever estimation of the *in vivo Leishmania* parasite doubling time in human of 7.8 days, which was only slightly slower than previously reported *in vitro* intracellular *L*. *donovani* amastigote replication rates within macrophages, which correspond to parasite doubling times of 4.2 and 2.9 days [35,36]. However, one limitation of this analysis is the lack of data on ‘natural’ parasite growth prior to initiation of treatment. Parasite growth was estimated based on patient data after treatment (Fexi10D), where the estimated parasite growth might have been affected by the treatment itself or selection of certain parasite subpopulations due to the treatment. Moreover, identification of parasite growth is also dependent on the sampling scheme, and parasite growth rate might be variable among patients, depending on host-related factors such as the activity of the immune system.

The unusual PK characteristics of liposomal amphotericin B, complicate the construction of a PK/PD model for this particular drug, since it is unknown to what extent systemic liposomal amphotericin B concentrations (or dose kinetics) are actually driving parasite clearance. Liposomal amphotericin B is preferentially taken up by blood monocytes and tissue macrophages, in which *Leishmania* parasites primarily reside. This assumption, in combination with the absence of monotherapy data for both liposomal amphotericin B and SSG, may have contributed to the minor underprediction of parasite clearance observed in the AmB-based regimens.

The combination regimens liposomal amphotericin B with SSG or miltefosine led to rapid drug-induced parasite clearance, resulting in undetectable blood parasite loads by the end of drug exposure in most patients (70–78% of patients had a parasite load <10 parasites/mL on Day 28). Miltefosine monotherapy resulted in a slower or delayed onset of parasite clearance, in line with the slow accumulation of miltefosine in the first week of treatment. Miltefosine treatment outcome has been shown to be associated with the time above a target concentration [37]. On the other hand, the long half-life of the drug results in adequate above-target exposure, which extends for a considerable period of time after the end of the 28-day monotherapy (MFC28D and MFA28D) and results in complete parasite clearance by Day 56 (parasite load <10 parasites/mL on Day 56 in 74–86% of patients). Fexinidazole treatment resulted in only partial drug-induced parasite clearance in most patients, with only 2/13 patients having complete parasite clearance by the end of treatment, as measured by qPCR. The results of this model suggest that adequate parasite clearance by the drug is important for achieving long-term clinical cure, as almost all patients on fexinidazole therapy who had incomplete drug clearance (by the treatment) failed therapy. This was confirmed by the ROC analysis, as the optimal blood parasite load cutoff values for classifying clinical relapse were around 10 parasites/mL, meaning that very low parasite levels by the end of treatment are already associated with higher risk of clinical relapse. This was in line with previous results of a descriptive analysis of these data, concluding that the blood parasite load on Day 56 was the best time to predict clinical outcome, with a comparable optimal cutoff value of 20 parasites/mL [11].

Parasite suppression after treatment, presumably driven by the host immune system, was empirically described in the model by an E_max_ function with variable onset and magnitude of parasite clearance. Parasite dynamics after treatment were highly variable among patients. The model suggested that patients with complete parasite suppression had a fast onset of the host immune response, occurring relatively rapidly after end of treatment, while patients with parasite recrudescence somewhere during follow-up had a late onset or weak magnitude of the immune response. Although we used a sophisticated analysis method to incorporate the complex interplay between parasites, drugs, and host, the haematological and biochemical data explored could not explain the between-patient variability in onset and magnitude of post-treatment suppression or, conversely, recrudescence of parasites.

Because of the multitude of effects that play a role in parasite dynamics, such as parasite growth, simultaneous parasite clearance by the treatment, and parasite suppression by the immune system, simultaneous estimation of all parameters led to over-parameterization of the model. However, by estimating the parasite growth rate, the drug effects, and the host immune effect separately based on representative subsets of the data, we could adequately estimate a parasite growth rate and drug effects for all drugs. Precise parameter estimates of the immune response model were, nevertheless, difficult to obtain. To improve the stability and the convergence of the model, various estimation methods were attempted, as well as implementing a mixture model with different k_imm_’s for cured and relapsed patients, but this did not result in stable parameter estimates. In future studies, more frequent blood sampling, especially during the follow-up phase, in combination with the identification of relevant host biomarkers, might improve the characterization of host suppression of the parasite load after treatment. While re-infection cannot be formally excluded, relapse is more likely as a cause of VL recurrence within the time-frame of the conducted clinical trials [38].

To predict parasite response after treatment, attempts were made to identify biomarkers for the activity of the immune system, by explaining variability in k_imm_, but none could be identified. Identification of a biomarker for activity of the immune system is complicated because there are many factors involved. Cytokines and other direct immunological biomarker measurements were not available from the clinical trials currently included. White blood cell count was evaluated as a predictor for the activity of the host immune system, but was not relevant. In cured patients, lymphocyte and albumin levels were higher and total protein levels were lower, although these results should be interpreted with caution as they were only available for a small proportion of patients (Fig C in S1 File). Depletion of lymphocytes and albumin is a symptom of VL infection, and could therefore reflect severity of disease, and subsequently the ability of the patient to clear the parasite. Moreover, lymphocyte count might directly reflect the function of the immune system, as a Th1 response is probably responsible for activation of infected macrophages that can lead to intracellular *L*. *donovani* killing [39], once there are enough active T cells [10]. It would be worthwhile to collect blood cell counts and albumin levels in patients during treatment and follow-up in future clinical trials, as well as exploring other biomarkers related to the immune response, to further investigate this relationship using the developed PK/PD modeling framework.

Individual model-based predictions of parasite AUC and parasite loads at different time points were compared to the final clinical outcome. Parasite load at start of treatment was not related to relapse, indicating that the severity of infection at start of treatment is not indicative for the final treatment outcome. Parasite AUC was not predictive for clinical outcome either, which might be due to the aforementioned differences in pharmacokinetics between the treatment regimens. For example, the onset of parasite clearance due to miltefosine is much slower due to the initial slow accumulation of miltefosine, resulting in higher AUCs until Day 10, 28, and 56 for the miltefosine monotherapies compared to combination therapies with liposomal amphotericin B, but this treatment nevertheless resulted in a comparable final outcome. The parasite load on Day 28 and Day 56 after start of treatment was related to relapse, indicating that both an adequate drug response and an early onset of immune system activation are needed. Although the blood parasite load on Day 28 or Day 56 could not correctly predict relapse in all individual patients (26% and 24% of relapsed patients were classified as cured based on the ROC results, respectively), the fraction of patients having a blood parasite load <10 parasites/mL by the end of treatment in a group of patients receiving the same VL treatment is an indication of the efficacy of the treatment in this population. This might be useful in clinical trials, where new dosing regimens or new combinations will be tested, to get an early indication of the long-term efficacy of this treatment, which would benefit from increased molecular biology capacity building in VL endemic areas in Eastern Africa. In the future, optimization of qPCR as a parasitological marker, possibly combined with other host biomarkers, could improve the sensitivity/specificity of a predictor for VL relapse, and be used as an endpoint in future VL trials to reduce the need for long periods of follow-up.

This is the first time that the complex mechanisms of parasite replication, treatment effects, and host response have been integrated into a comprehensive semi-mechanistic model, which provides insight into the *in vivo* parasite growth rate and parasite clearance rates by different drugs. The model could serve as a framework for optimization of VL treatment regimens, and blood parasite load at the end of treatment could be a useful biomarker to assess efficacy of treatment regimens in clinical trial settings.

## Supporting information

S1 File Supplementary data. (DOCX)
