# Supplementary material for: Leishmania blood parasite dynamics during and after treatment of visceral leishmaniasis in Eastern Africa: A pharmacokinetic-pharmacodynamic model
Source: PLoS Negl Trop Dis. 2024 Apr 19;18(4):e0012078. doi: 10.1371/journal.pntd.0012078 (PMC11062534; doi:10.1371/journal.pntd.0012078)

**Supplementary Data**

**Table A.** Number of patients in defined typical blood parasite loads profiles after treatment, including 1) complete parasite clearance with no parasite recrudescence during follow-up (complete parasitological cure) 2) initial parasite clearance followed by parasite recrudescence, where parasite regrowth is initiated at different time points during the follow-up period, and 3) initial parasite clearance followed by parasite recrudescence early after treatment, followed by parasite clearance later during follow-up. Patients with only one observation or with no/little parasite clearance are classified in the NA group.

|  | **Typical blood parasite loads profiles after treatment (n)** | | | |
| --- | --- | --- | --- | --- |
| **Treatment regimen** | **Profile 1** | **Profile 2** | **Profile 3** | **NA** |
| **A. LEAP0208 AmB+SSG10D** | 26 | 5 | 8 | 1 |
|  | (ID 307, 311,314, 319,321, 329, 340, 342, 345, 348, 352, 354, 357, 359, 505, 510, 516, 519, 521, 525, 536, 538, 547, 553, 557, 715) | (ID 302, 513, 533, 540, 709) | (ID 317, 338, 360, 530, 544, 704, 707, 714) | (ID 325) |
| **B. LEAP0208 AmB+MF10D** | 19 | 14 | 8 | 3 |
|  | (ID 304, 313, 322, 327, 331, 333, 334, 336, 341, 349, 351, 362, 365, 511, 514, 522, 524, 531, 718) | (ID 306, 323, 346, 356, 506, 529, 537, 539, 546, 548, 549, 556, 706, 708) | (ID 309, 316, 363, 517, 532, 702, 712, 717) | (ID 367, 368, 374) |
| **C. LEAP0208 MF28D** | 21 | 15 | 4 | 6 |
|  | (ID 301, 308, 315, 320, 324, 328, 335, 337, 339, 344, 353, 364, 369, 515, 520, 528, 535, 541, 542, 554, 701) | (ID 303, 312, 332, 355, 358, 361, 512, 518, 527, 545, 552, 555, 705, 711, 716) | (ID 343, 350,703, 713) | (ID 318, 366, 523, 526, 551, 710) |
| **D. LEAP0714 MF28D** | 12 | 7 | 9 | 1 |
|  | (ID 606, 607, 608, 609, 904, 906, 907, 909, 910, 913, 916, 918) | (ID 602, 604, 905, 908, 915, 919, 921) | (ID 601, 603, 901, 902, 903, 911, 912, 917, 920) | (ID 914) |
| **E. FEXI-VL-001 Fexi10D** | 2 | 10 | 0 | 1 |
|  | (ID 1,8) | (ID 3, 4, 5, 6, 7, 9, 10, 11, 12, 13) |  | (ID 2) |

**Fig A.** Individual model-based predictions of blood parasite loads per treatment arm.

A. LEAP0208 AmB+SSG10D


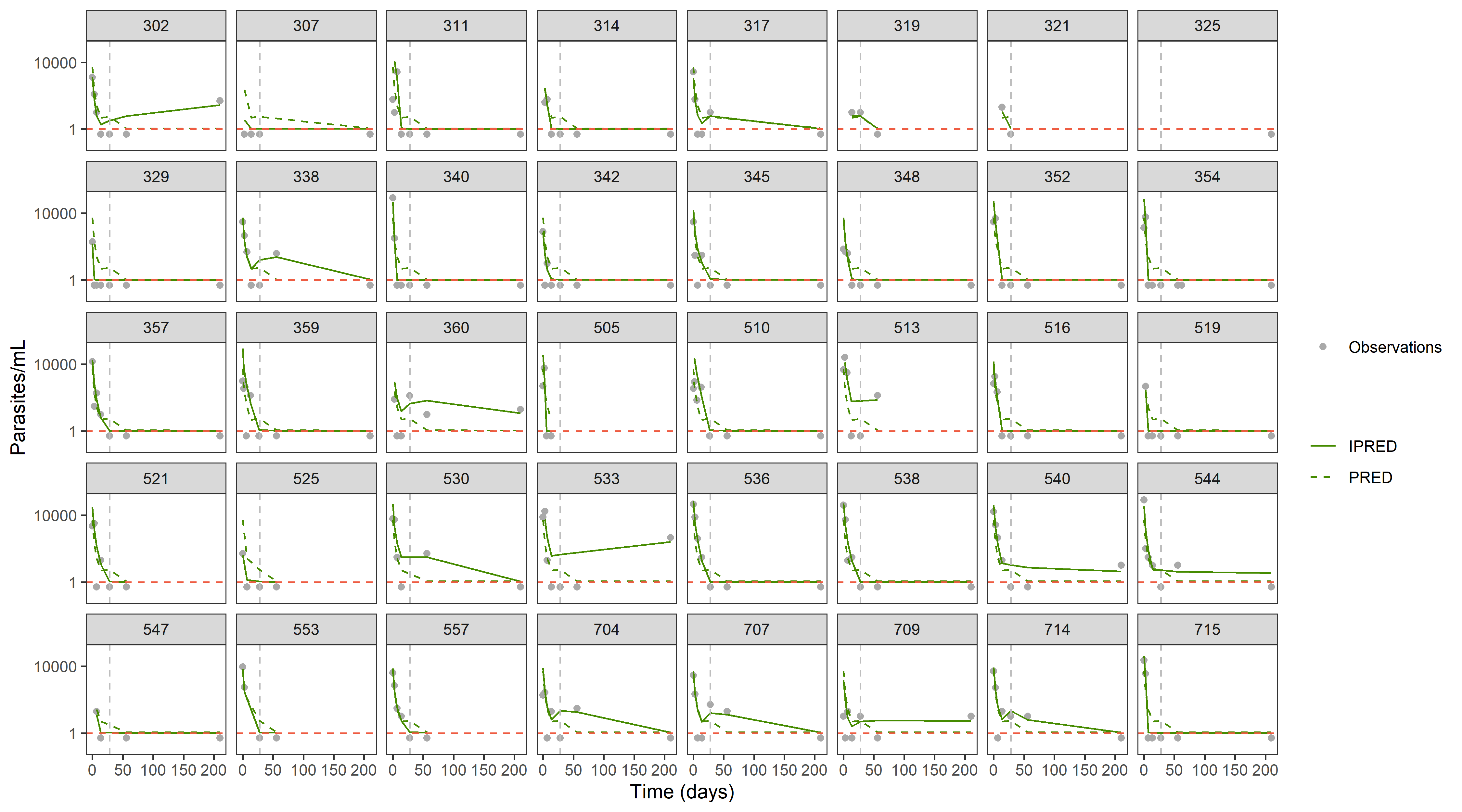


B. LEAP0208 AmB+MF10D


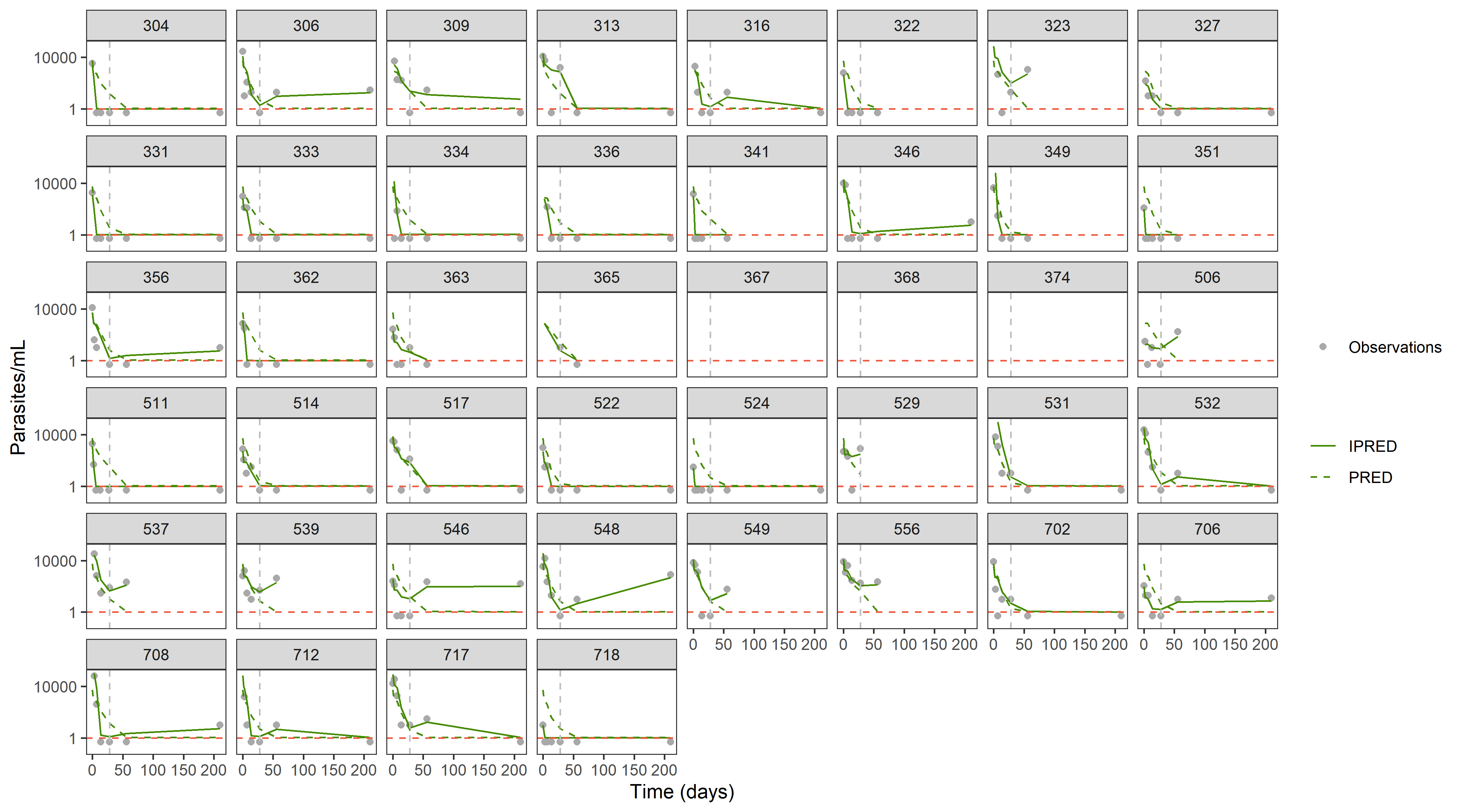


C. LEAP0208 MF28D


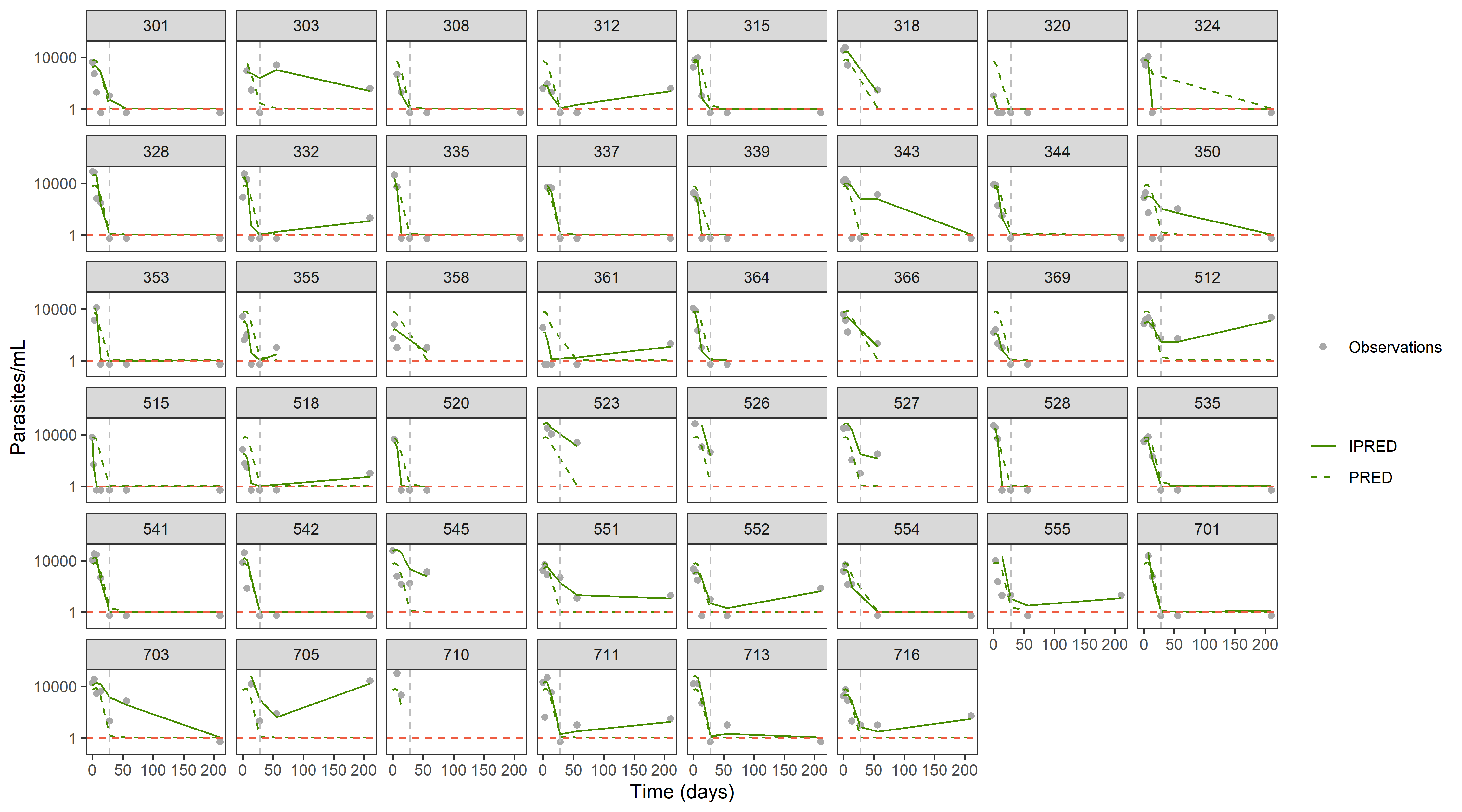


D. LEAP0714 MF28D


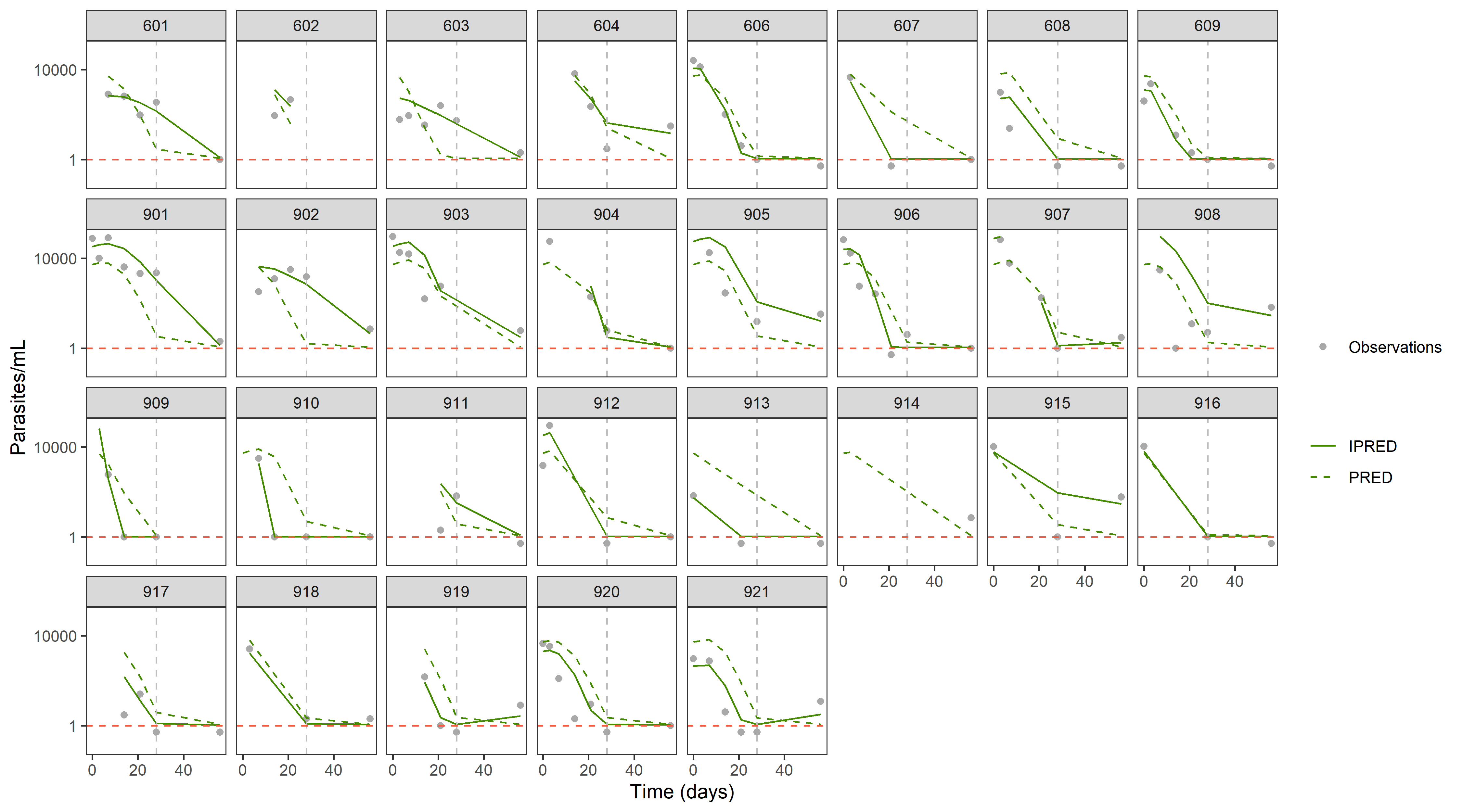


E. FEXI-VL-001 Fexi10D


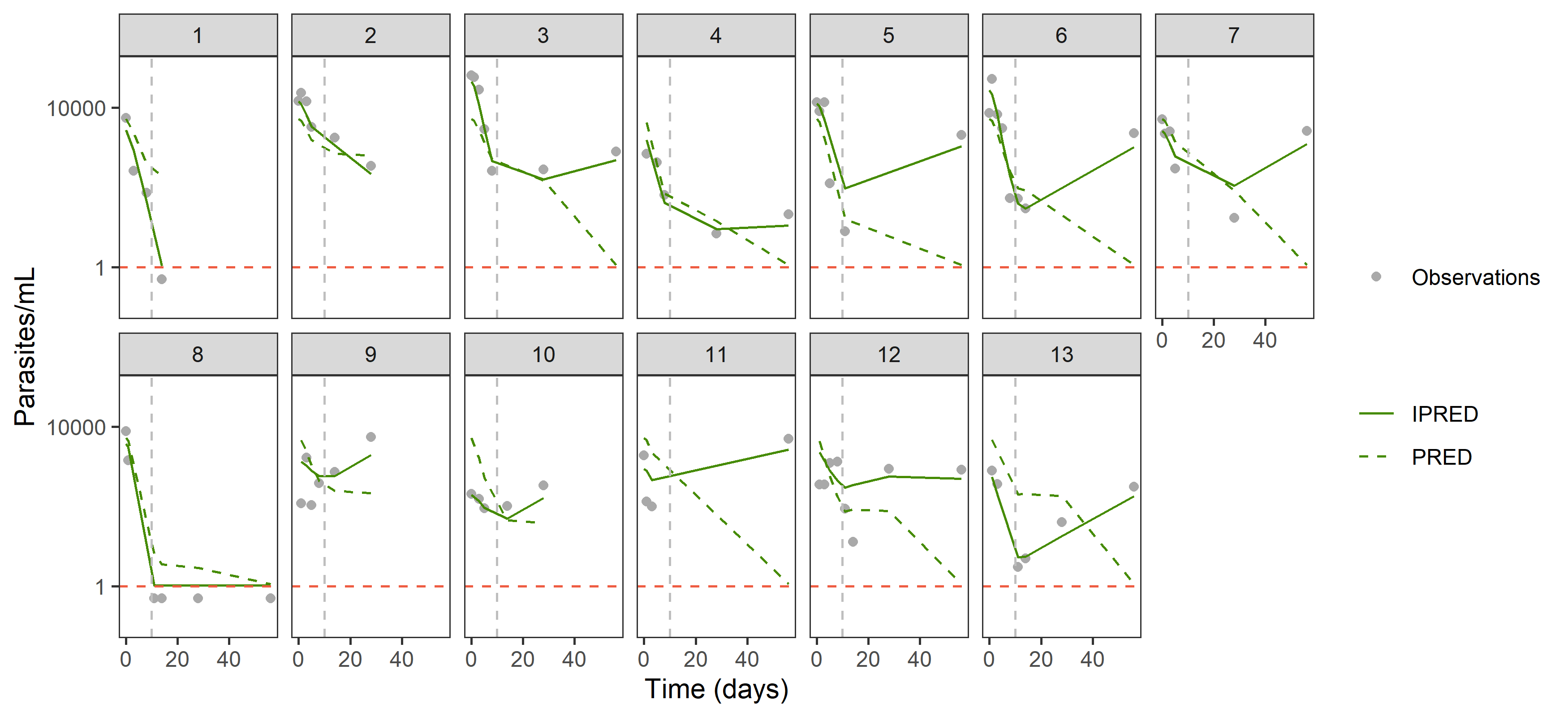


**Fig B.** Individual model fits in a selection of patients presenting different parasite profiles. Grey dots: observed blood parasite loads; dashed green line: population predictions; solid green line: individual predictions; dashed red line: lower limit of quantification; dashed grey line: end of treatment.

**
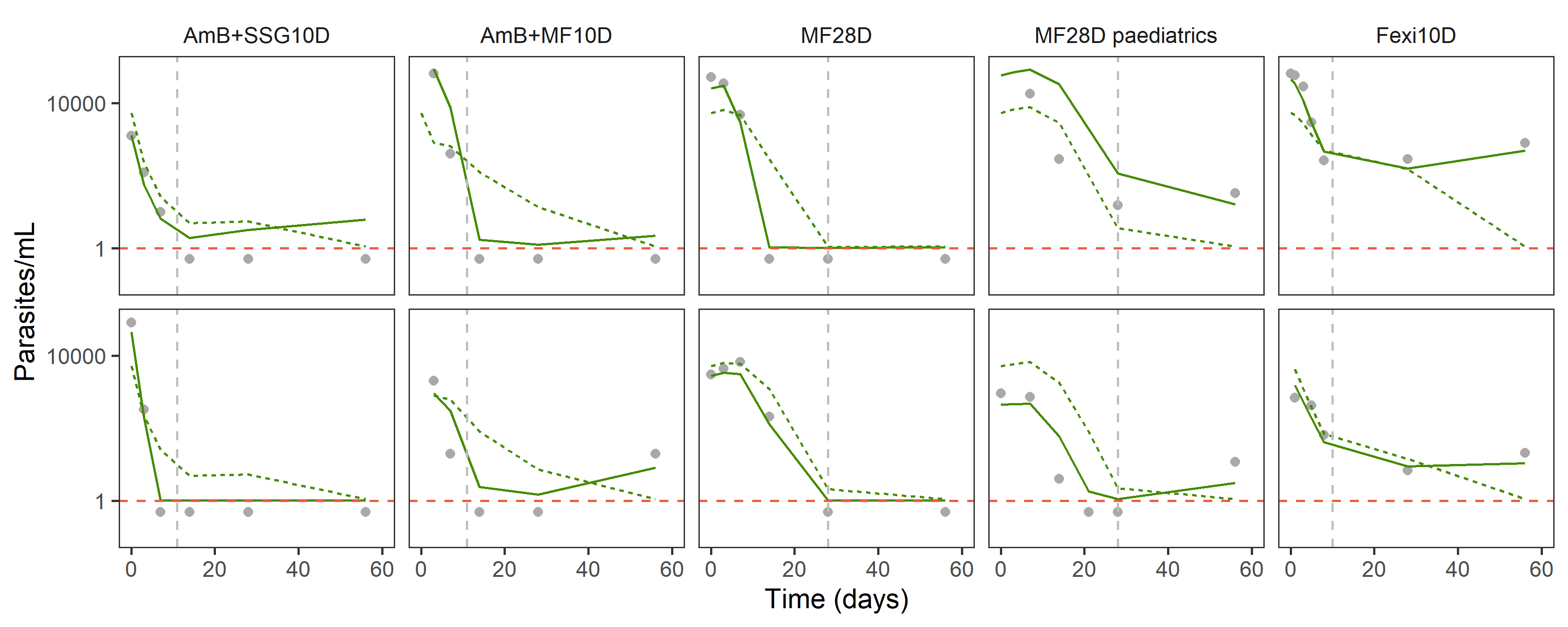
**

**Fig C.** Individual model fits of parasite loads during and after treatment in a selection of patients receiving different treatment regimens. Grey dots: observed blood parasite loads; dashed green line: population predictions; solid green line: individual predictions; dashed red line: lower limit of quantification; dashed grey line: end of treatment.


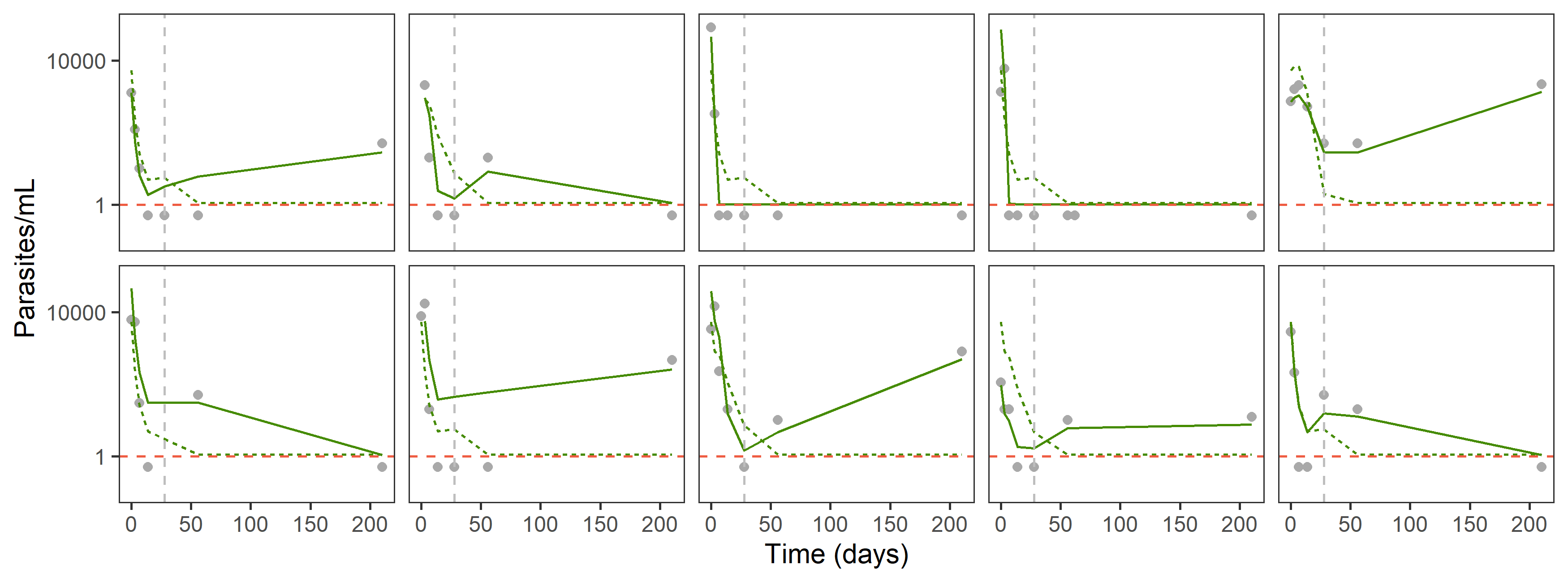


**Fig D.** Hematological data **A.** Hematological data available of all studies, colored by parasitological response.


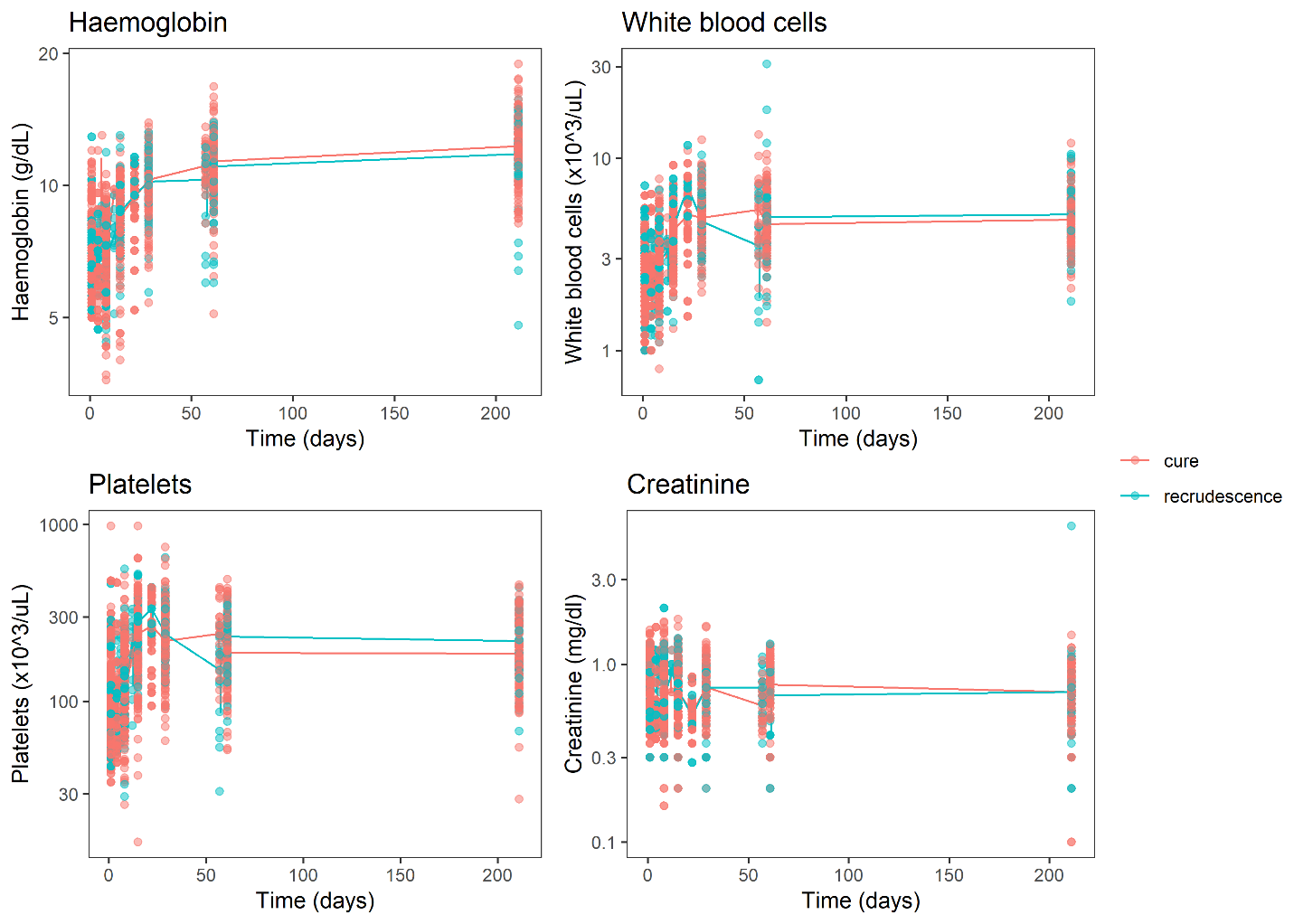


B**.** Hematological data available of LEAP0714 and FEXI-VL-001, colored by parasitological response.


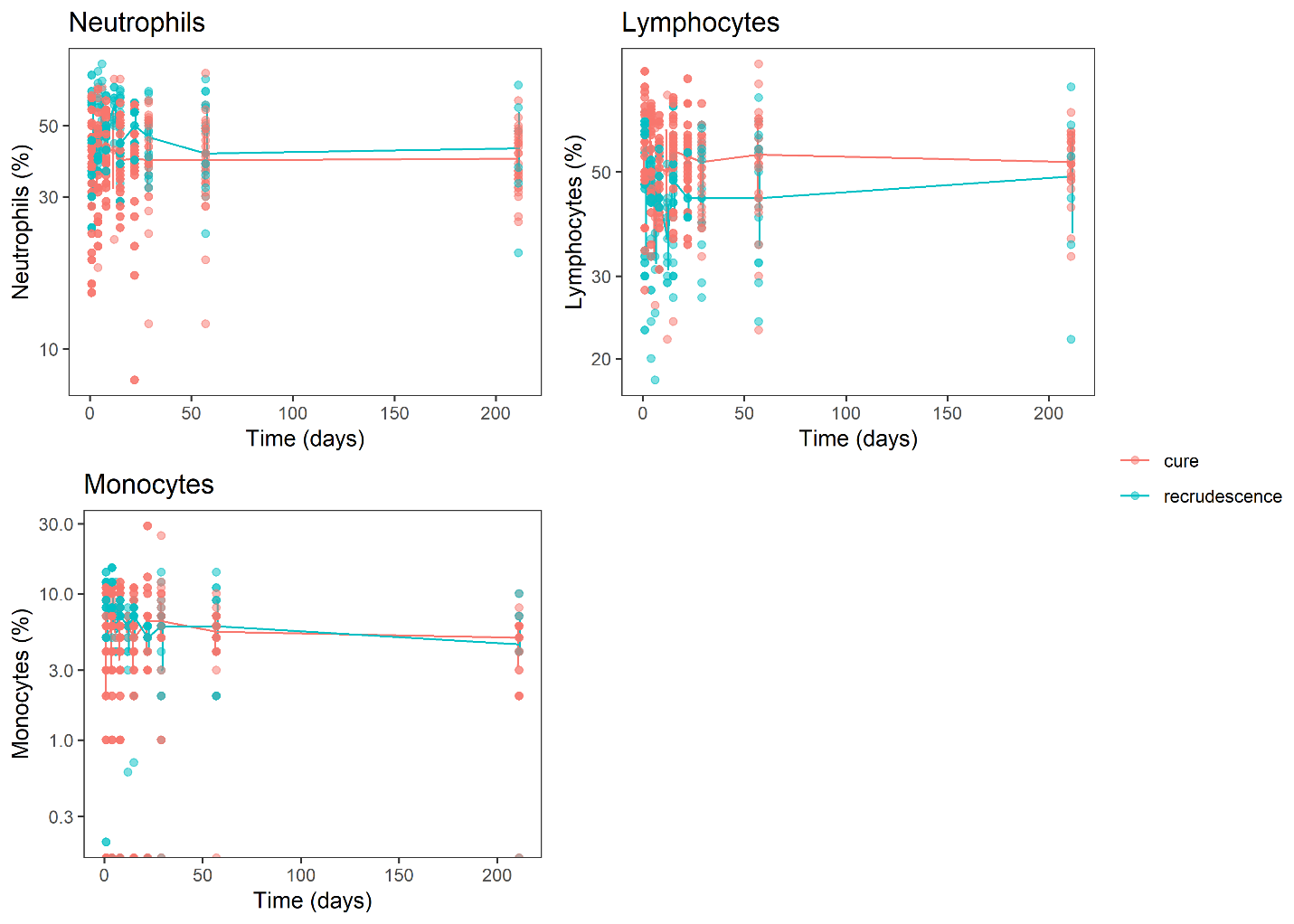


C. Hematological data available of FEXI-VL-001, colored by parasitological response.


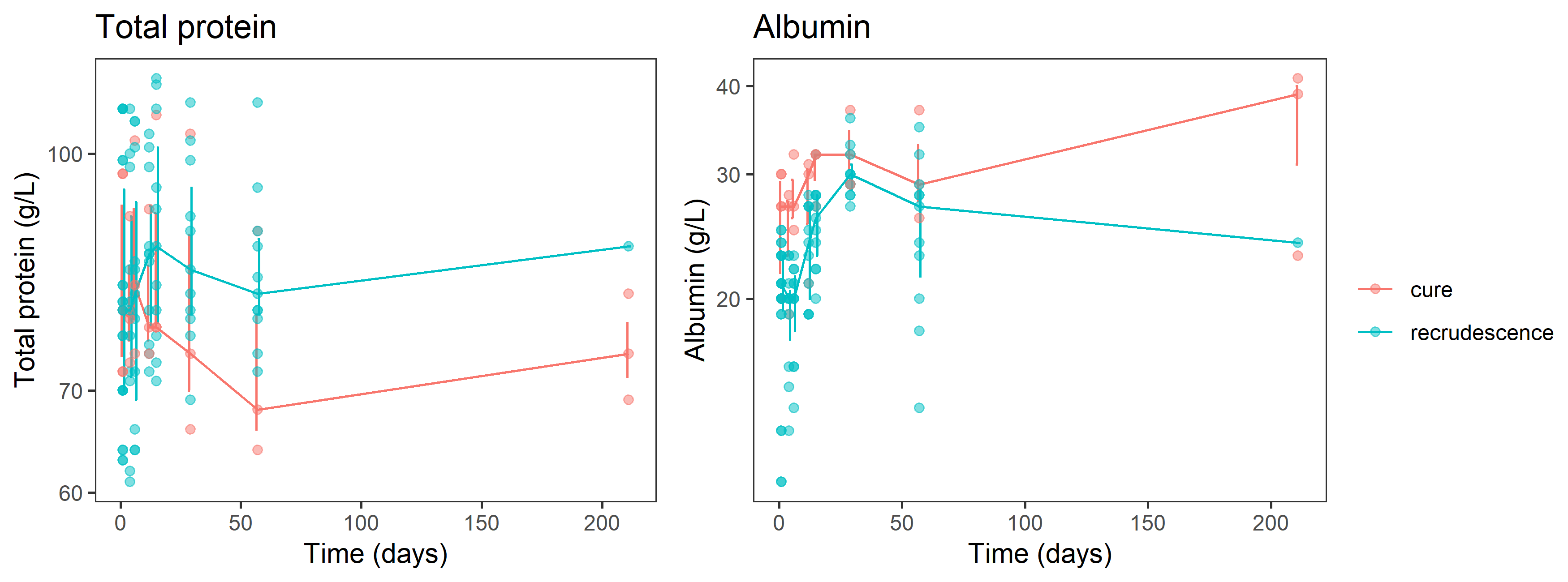

Supplement: S1 File — (DOCX) [file pntd.0012078.s001.docx]
